# The RNA-mediated, asymmetric ring regulatory mechanism of the transcription termination Rho helicase decrypted by time-resolved Nucleotide Analog Interference Probing (trNAIP)

**DOI:** 10.1093/nar/gku595

**Published:** 2014-07-12

**Authors:** Emilie Soares, Annie Schwartz, Marcello Nollmann, Emmanuel Margeat, Marc Boudvillain

**Affiliations:** 1 Centre de Biophysique Moléculaire, CNRS UPR4301, rue Charles Sadron, 45071 Orléans cedex 2, France; 2 CNRS UMR5048, Universités Montpellier I et II, Centre de Biochimie Structurale, 29 rue de Navacelles, 34090 Montpellier, France; 3 INSERM U1054, Montpellier, France; 4 ITP Sciences Biologiques & Chimie du Vivant, Université d'Orléans, France

## Abstract

Rho is a ring-shaped, ATP-dependent RNA helicase/translocase that dissociates transcriptional complexes in bacteria. How RNA recognition is coupled to ATP hydrolysis and translocation in Rho is unclear. Here, we develop and use a new combinatorial approach, called time-resolved Nucleotide Analog Interference Probing (trNAIP), to unmask RNA molecular determinants of catalytic Rho function. We identify a regulatory step in the translocation cycle involving recruitment of the 2′-hydroxyl group of the incoming 3′-RNA nucleotide by a Rho subunit. We propose that this step arises from the intrinsic weakness of one of the subunit interfaces caused by asymmetric, split-ring arrangement of primary RNA tethers around the Rho hexamer. Translocation is at highest stake every seventh nucleotide when the weak interface engages the incoming 3′-RNA nucleotide or breaks, depending on RNA threading constraints in the Rho pore. This substrate-governed, ‘test to run’ iterative mechanism offers a new perspective on how a ring-translocase may function or be regulated. It also illustrates the interest and versatility of the new trNAIP methodology to unveil the molecular mechanisms of complex RNA-based systems.

## INTRODUCTION

Many key biological processes, such as replication, transcription or virus packaging, involve the translocation of nucleic acids (NAs) by NTP-dependent molecular motors. These motors often adopt a ring-shaped oligomeric structure encircling the NA substrate (1–3). This configuration is thought to increase the processivity of ring-shaped motors but constrains their mechanisms of action in ways that remain insufficiently understood. Salient issues include how NTP hydrolysis sites (located at subunit interfaces) and NA binding sites (within the central ring channel) are allosterically coupled within the oligomeric ring, what molecular events compose the motor cycle and to what extent these elementary features are conserved among ring-shaped translocases.

**Figure 1.**
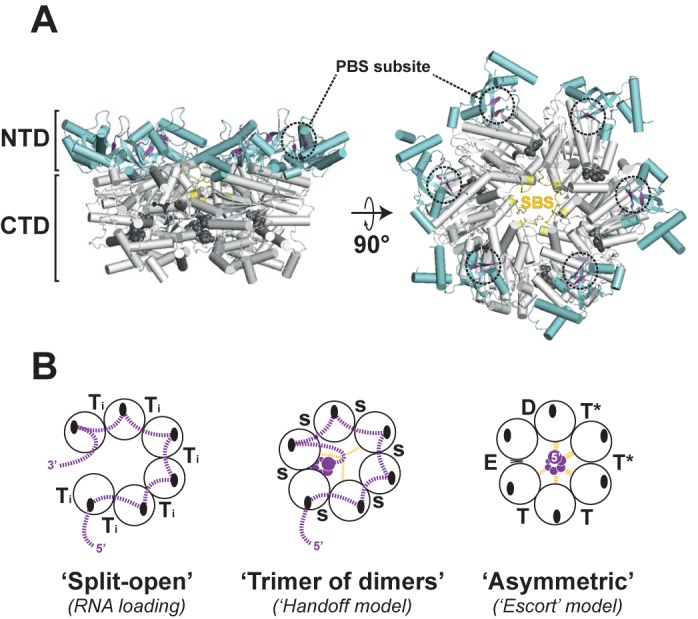
**(A)** Structure of the Rho hexamer (PDB: 3ICE) with N- and C-terminal domains (NTD and CTD, respectively) in cyan and white, respectively. PBS and SBS residues are shown in magenta and yellow, respectively. ADP.BeF_3_ analogs in ATPase pockets at subunit interfaces are shown in black sphere representation. **(B)** Schematics of the three structural states resolved by crystallography (7,13,14) are shown with small, black ovals representing subunit PBS subsites. Dotted lines in magenta depict the putative trajectory of the RNA chain to, between and from crystal-resolved RNA binding sites (RNA residues bound to the SBS are depicted as spiraling magenta circles). Ti indicates AM-PNP bound to an ATPase site in a non-productive conformation (in PDB #1PVO); s represents a sulfate ion in an empty ATPase site (in PDB #2HT1). ADP.BeF_3_ analogs are bound to ATPase sites in a nucleotide exchange state (E), ATP-bound state (T), hydrolysis-competent state (T*) or product state (D) in PDB #3ICE.

Transcription termination factor Rho is a model ring-shaped, adenosine triphosphate (ATP)-dependent [5′→3′] RNA translocase that contributes to the regulation and protection of bacterial genomes by mediating the dissociation of transcription elongation complexes and/or unwinding of RNA:DNA hybrids (R-loops) (4,5). In contrast to many other ring-shaped translocases, Rho does not need a specific cofactor (ring loader) to load onto its substrate. Rather, the Rho hexamer contains N-terminal RNA binding motifs that form a composite, crown-like Primary Binding Site (PBS) on top of the Rho ring (Figure 1A) (6,7). The PBS serves to capture single-stranded, cytosine-rich RNA and to guide it into the central Rho channel, presumably by a mechanism involving a ‘split-open’ ring state **(**Figure 1B) as that observed by electron microscopy (8) or in a subset of crystal structures of Rho (7,9). Within the central channel, a second group of Rho residues compose the Secondary Binding Site (SBS; Figure 1A) responsible for enzymatic activation (10–12) and ATP-dependent translocation of RNA (13,14). Importantly, RNA:SBS contacts are thought to promote closed state(s) of the Rho ring as observed in two recent crystal structures (13,14). However, the organization of the Rho hexamer, either as a ‘trimer of dimers’ or as an asymmetric toroid (Figure 1B) as well as the identity of putative SBS residues differ in both structures and led to two distinct models for RNA translocation.

In the first model, ATP-dependent interconversion of protomers in the ‘trimer of dimers’ mediates movement of two triplets of Lys283 ‘levers’ pulling on RNA within the central channel (13). Downstream RNA, which forms additional backbone- and base-specific contacts in a subunit interface cleft (Figure 1B), is also sequentially transferred from one interface to the next upon protomer interconversion (RNA ‘handoff’ model). Moreover, crystallographically resolved RNA dinucleotides in the six PBS clefts of the ‘trimer of dimers’ structure suggest that the RNA chain is not released from the PBS during translocation (Figure 1B) (13). This is consistent with biochemical (15) and single-molecule experiments ((16); our unpublished observations) supporting a ‘tethered tracking’ mechanism in which an RNA loop develops between the SBS (where RNA contacts change during translocation) and PBS (where RNA contacts are maintained throughout) anchor points.

In the second translocation model, each Rho subunit escorts RNA through the central channel by sequentially adopting one of the six distinct subunit conformations found in the asymmetric ring structure (14). At any time, six nucleotides of single-stranded RNA are maintained in a tight spiral staircase within the central channel. Only these nucleotides are bound to the Rho subunits via SBS contacts to RNA backbone components (Figure 1B). This subunit ‘escort’ model, which is analogous to that proposed for the ring-shaped DNA helicase E1 (17), predicts that Rho translocates RNA in monotonous steps of one nucleotide (nt) per molecule of hydrolyzed ATP (14).

Recent probing of putative SBS residues by mutagenesis supports the ‘asymmetric’ ring structure (18). Yet, the structure may not fully recapitulate important RNA translocation features. For instance, the short r(U)_12_ ligand used for crystallization and empty PBS clefts in the structure (14) cannot illustrate the persistence of PBS:RNA contacts and formation of an RNA loop between the PBS and SBS during translocation (15,16). Aspects pertinent to this ‘tethered tracking’ mechanism, such as putative cross-talk between the RNA-bound PBS and SBS (18), are thus not addressed by the ‘escort’ model. This model also does not explain the large, 2′-hydroxyl-dependent step size of ∼7-nt that has been determined for the Rho helicase from periodic chemical interference patterns obtained upon probing Rho translocation and unwinding activity with a Nucleotide Analog Interference Mapping (NAIM) assay (19). Thus, features governing the strict RNA specificity of the Rho motor (20) and how they contribute to the Rho translocation cycle remain largely uncertain.

To clarify these essential features, we first compared NAIM penalty profiles obtained upon Rho-directed unwinding of RNA:DNA substrates having distinct architectures and compositions. Next, we explored the ‘chemo-kinetic’ framework (i.e. kinetic importance and role of individual atoms/functional groups) of the Rho helicase in depth using a new, time-resolved Nucleotide Analog Interference Probing (trNAIP) assay. Our data show that Rho translocates RNA residues non-uniformly irrespective of the substrate identity and support structural coupling between the PBS and SBS during translocation. In addition, we show that the 2′-hydroxyl-dependent stepping behavior of Rho (19) is due to the cyclic formation of a dissociation-prone translocation intermediate. The lack of a 2′-hydroxyl moiety at a critical, in-register position of the RNA track is sufficient to destabilize this intermediate and to significantly increase the probability of dissociation of the Rho:RNA complex. Destabilization is greater if the 2′-OH moiety is replaced by a 2′-OCH_3_ group rather than by a 2′-H or 2′-F atom and is modulated by RNA sequence. Altogether, these data show that the Rho:RNA interaction network varies significantly during translocation and becomes critically dependent on 2′-OH contact(s) and steric constraints at periodic intervals. We propose a new, comprehensive model for Rho translocation that accounts for this and most other information. Overall, our work reconciles conflicting data on Rho mechanisms, reveals principles for the functioning and regulation of a model ring-shaped translocase that are exquisitely linked to its molecular architecture, and illustrates the remarkable potential of the new trNAIP methodology to dissect the mechanisms of dynamic RNA-based systems.

## MATERIALS AND METHODS

### Chemicals, enzymes and NA substrates

Chemicals and oligonucleotides were purchased from Sigma-Aldrich and Eurogentec, respectively. The rNTPs and analogs (NTPαS, 2′-deoxyNTPαS, 2′-OMe-ATPαS, 2′-OMe-UTPαS and 2′-F-ATPαS) were from Promega and Glen Research, respectively. The 2′-OMe-CTPαS and 2′-OMe-GTPαS analogs were obtained from Biolog-LSI (Germany). The Rho protein from *Escherichia coli* (amino acids numbers refer to this protein throughout the paper) and Poly[rC] fragments (>300 nts) were prepared and purified as described previously (21). Double-stranded DNA templates were prepared by standard polymerase chain reaction (PCR) amplification of synthetic DNA templates, as detailed elsewhere (22). Both unmodified and analog-containing RNA strands were prepared by *in vitro* transcription of the resulting DNA templates with wild-type (WT) or Y639F mutant of T7 RNA polymerase. Conditions for *in vitro* transcriptions (nucleotide concentrations; WT or mutant polymerase) were adjusted as described previously (19,22,23) to yield analog incorporation levels of ∼5% (24). All RNA and DNA strands were purified by denaturing polyacrylamide gel electrophoresis (PAGE) and stored at −20°C in M_10_E_1_ buffer (10 mM MOPS, pH 6; 1 mM ethylenediaminetetraacetic acid (EDTA)). The bipartite and tripartite RNA:DNA substrates were prepared from individual components (one of which contained a ^32^P-end label) by heat denaturation/renaturation and PAGE purification protocols as described previously (18,19). Substrates were stored at −20°C in helicase buffer (20 mM HEPES, pH 7.5, 0.1 mM EDTA and 150 mM sodium glutamate [buffer I] or potassium acetate [buffer II]). Concentrations of RNA/DNA and substrate stocks were determined from their absorbance at 260 nm measured with a μl-spectrophotometer (Nanodrop).

### NAIM experiments

NAIM experiments with NαS- (or 2′-deoxyNαS-, 2′-F-AαS-, or 2′-OMeNαS-) modified substrates were performed as described previously (19,22). Briefly, the RNA substrates (5 nM, final concentration) were mixed with 4 molar equivalents of Rho hexamers in helicase buffer (buffer I for substrate A; buffer II for substrates B and C) and incubated for 3 min at 30°C. Then, Mg-ATP (1 mM, final concentration), and DNA trap (400 nM, final concentration; the DNA trap is complementary to the D^57A^ oligodesoxyribonucleotide released upon duplex unwinding) were added before further incubating the helicase mixture at 30°C. Four volumes of quench buffer (27 mM EDTA, 0.7% (w/v) sodium dodecyl sulphate (SDS), 4% (w/v) Ficoll-400) were added to mixtures at times corresponding to ∼20% of reaction extent (19). The ^32^P-labeled products were separated and purified on 7.5% PAGE gels that contained 0.5% (w/v) SDS. The phosphorothioate linkages in the transcripts were cleaved by treatment with 1 mM iodine for 3 min at 37°C. The resulting cleavage products were resolved by denaturing PAGE (we used 9–20% PAGE gels to resolve different RNA sizes) and quantified with a Typhoon Trio imager and ImageQuant TL v8 software (GE-healthcare) using the full, undistorted dynamic range of the gel scans (gamma settings were adjusted only for gel visualization). Analysis of NAIM experiments was performed as described previously by converting raw NAIM signals obtained from gel band ratios into normalized λ interference factors (19,22,23).

### trNAIP experiments

Preliminary trNAIP experiments indicated that selection of reaction species by PAGE, as in NAIM, was a method bottleneck plagued by poor recovery of low-abundance species (e.g. unwound species at the reaction onset) upon elution from gel bands (data not shown). These issues were addressed by opting for affinity bead partitioning as follows. For each trNAIP experiment, about 0.4 mg (40 μl) of streptavidin-coated magnetic beads (Dynabead M-280, Invitrogen) were washed thrice with 80 μl of BW buffer (1 M KCl, 5 mM Tris-Cl, pH 7.5, 0.5 mM EDTA) before incubation with 4 pmoles of biotinylated RNA:DNA substrate in 80 μl of helicase buffer II for 1 h at 18°C. Beads were then separated from supernatant, washed twice with 80 μl of buffer II, and incubated for 10 min at 25°C with 17.8 pmoles of Rho hexamer in 220 μl of buffer III (helicase buffer II supplemented with 0.1 mg/ml of acetylated bovine serum albumin). After elimination of the supernatant containing unbound Rho hexamers, the helicase reaction was initiated by addition of 220 μl of pre-warmed buffer III containing Mg-ATP (1 mM, final concentration), poly[rC] (3 μM in rC residues) and non-biotinylated DNA competitor (same sequence as biotinylated DNA strand, 800 nM, final concentration). The reaction mixture was incubated at 25°C and shacked at 300 revolutions per minute in a thermomixer (Eppendorf) to homogenize the bead suspension. Suspension aliquots (20 μl) were removed at various times, mixed with 1 volume of quench buffer (40 mM EDTA, 1 μM DNA competitor), and kept on ice before separation of the bead (B1) and supernatant (S1) fractions (0.5 μl of each suspension aliquot was directly analyzed on a control 8% PAGE gel containing 0.5% SDS). Beads from the B1 fractions were mixed with 40 μl of DB buffer (50% formamide, 0.75% SDS, 1.25 μM DNA competitor, 4 mM MOPS pH 6, 0.4 mM EDTA) and incubated for 2 min at 72°C. Supernatant (S2) fractions were then quickly collected on a MagRack (GE Healthcare) that was pre-heated at 72°C. In this way, ∼100% of the ^32^P labeled RNA molecules present in B1 fractions were detached from the bead-bound biotinylated DNA strands and recovered in the S2 fractions. Buffer compositions of the S1 and S2 fractions were then equalized in order to avoid reactivity biases during subsequent treatment with iodine. Analog-containing transcripts were then cleaved with iodine and analyzed by denaturing PAGE as described in the preceding section. For each position of analog incorporation (i), a reaction progress curve was deduced from the fraction of RNA:DNA hybrids (*F*_*t*,*i*_) unwound at time *t* defined as: *F*_*t*,*i*_ = *I*(S1)*_i_*/(*I*(S1)*_i_* + *I*(S2)*_i_*), where *I*(S1)*_i_* and *I*(S2)*_i_* are the intensities of the bands corresponding to analog incorporation at position *i* in the S1 and S2 fractions, respectively.

## RESULTS

### Cyclic 2′-hydroxyl-dependency is a substrate-independent hallmark of the Rho helicase

**Figure 2.**
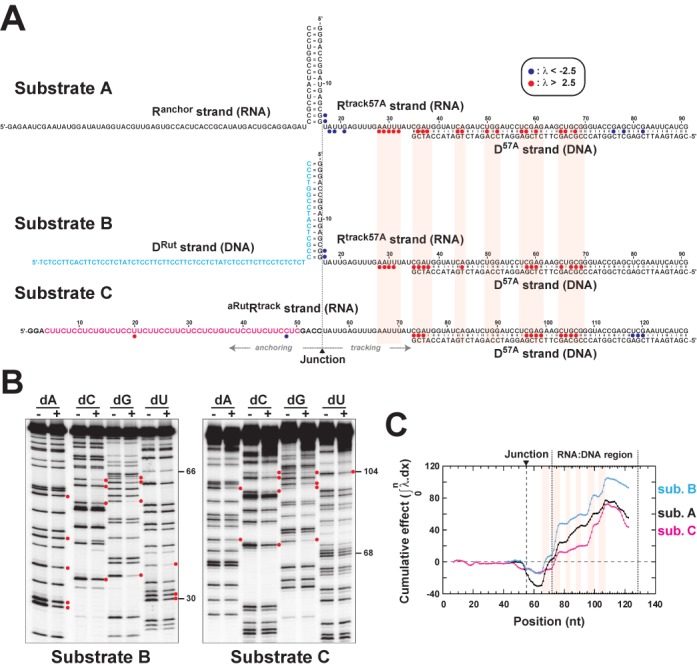
Standard NAIM probing of the helicase activity of Rho with ‘forked’ and ‘linear’ RNA:DNA substrates. Detrimental 2′-deoxy interference clusters observed in this study and in ref. (19) are outlined by light red boxes. **(A)** Composition of the RNA:DNA substrates used in the study. Blue and red circles locate the positions of 2′-deoxy NαS interference effects (key is inset) within the RNA ‘tracking’ strands of the substrates. Normalized λ discrimination factors depict detrimental (λ > 0) and favorable (λ < 0) effects with the same intensity scale and account for the standard deviations of individual data sets (19,22,23). Signals with |λ| > 2.5 fall outside the 98.8% confidence interval for random signal variations. **(B)** Representative sequencing PAGE gels showing 2′-deoxy NαS interference effects obtained upon Rho-directed unwinding of substrates B and C (see ref. (19) for substrate A). Red dots locate positions of bands corresponding to detrimental effects (λ > 2.5). Gamma settings were adjusted to facilitate visualization of interference signals. **(C)** Cumulative 2′-deoxy NαS interference curves obtained upon NAIM probing of the tracking RNA strands. Nucleotide numbering is that of substrate C.

The periodic, 2′-OH-dependent translocation behavior of the Rho helicase was uncovered with a NAIM assay (Supplementary Figure S1) and minimal 3-piece RNA:DNA substrates (e.g. substrate A in Figure 2A) (19). These substrates were designed to maximize NAIM signals but possess two distinctive features that influence their handling by Rho. First, they lack a cytosine-rich *Rut* (Rho utilization) site to stabilize the Rho:RNA complex during its formation and conversion into a catalytically active isoform (25) and, possibly, during ‘tethered tracking’ of Rho along RNA (15,16). Second, they contain an intervening RNA helix that restricts the conformational freedom of productive Rho:RNA complexes (Supplementary Figure S1, inset) (19). This intervening helix also splits RNA into two functionally distinct arms which are presumed to bind only to the PBS (‘anchoring’ arm) and SBS (‘tracking’ arm), respectively (19,22,26).

To assess if these distinctive features significantly affect 2′ hydroxyl-dependent translocation by the Rho helicase, we probed the 2′-OH moieties of two other RNA:DNA substrates using standard NAIM. Substrate B has the same 3-piece, ‘forked’ architecture than substrate A but contains a pyrimidine-rich, *Rut*-like sequence in its ‘anchoring’ arm, D^Rut^ (Figure 2A). Moreover, the D^Rut^ arm is made of DNA (Figure 2A, in blue) to ensure that only ‘tracking’ arm (R^track57A^) components can activate the RNA-dependent ATP hydrolysis and motor activities of Rho (26,27). Substrate C is a more classical, ‘linear’ RNA:DNA construct containing a synthetic *Rut* site (Figure 2A, in pink) upstream from the RNA:DNA hybrid region (28). Importantly, the three substrates differ only in their upstream ‘anchoring’ sections while they contain the same single-stranded RNA and RNA:DNA hybrid sequences downstream from a theoretical anchoring-tracking junction (Figure 2A). These differences are nonetheless sufficient to make substrates B and C significantly more reactive than substrate A which is unwound by Rho at a ∼20-fold lower rate (18).

Using our NAIM setup (Supplementary Figure S1), we compared the patterns of interference effects obtained after Rho-directed unwinding of substrates A, B and C (∼20% reaction extent in each case) that were modified randomly with a 2′-deoxy-NαS analog in their tracking RNA arm. For the three substrates, we observed significant 2′-deoxy-NαS interference effects (Figure 2A and B) that tend to cluster into peaks along the RNA track (Supplementary Figure S2). These specific interference patterns yield periodic, step-like increases in the corresponding ‘cumulative effect’ curves (Figure 2C) characteristic of the 2′-OH-dependent stepping behavior of the Rho helicase (19). Although interference positions located downstream from the anchoring-tracking junction largely coincide for the three substrates, the intensities of interference effects (Figure 2A and Supplementary Figure S2) and step-like ‘cumulative’ increases (Figure 2C) vary. Variations located upstream from the RNA:DNA helix may be attributed, at least in part, to structural constraints on the Rho:substrate complexes that are not identical at the start of the unwinding reaction (Supplementary Figure S3). Downstream variations suggest that substrate-specific constraints do not disappear during translocation and unwinding. Rather, it appears that the distinct molecular components preceding the anchoring-tracking junction in the three substrates (Figure 2A) somewhat modulate ‘at distance’ the response of Rho to chemical alterations of its RNA track. This supports that PBS:RNA contacts persist during Rho translocation (15,16) and, possibly, that PBS:RNA and SBS:RNA interactions are allosterically coupled (18).

Despite the differences mentioned above, positions of step-like increases in cumulative profiles (Figure 2C) as well as interference autocorrelation maxima (Supplementary Figure S2) are similar for the three substrates. These observations support a scenario where the translocating Rho goes through 2′-hydroxyl-dependent states at similar track positions for the three substrates even if the NAIM penalty landscape is affected by the distinct substrate architectures. Our extensive NAIM analysis (Figure 2 and Supplementary Figure S2) (19) thus shows that the periodic requirement for a 2′OH moiety during translocation (every ∼7 nt on average) is an inherent feature of the Rho helicase, independent of the architecture and composition of the substrate used.

### Probing Rho translocation mechanism with trNAIP

To determine the origin of the 2′-hydroxyl-dependent translocation events, we sought to quantitate the kinetic effects of chemical substitutions of the 2′-OH moiety for each position of the RNA track. Since the individual preparation and testing of tens of chemically modified RNA:DNA substrates by classical methods is impractical, we envisioned a new combinatorial approach, trNAIP, wherein reaction progress curves (named ‘kinetic traces’ hereafter) attributable to single substitutions of the 2′-OH moiety are compiled quickly from a succession of NAIM selections performed at different reaction times.

**Figure 3.**
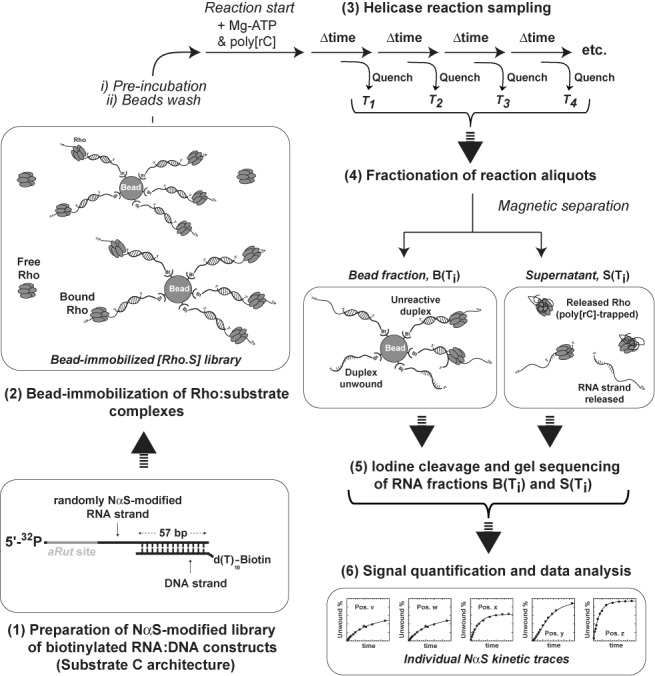
Principle of the trNAIP approach implemented to study Rho translocation/unwinding mechanisms. The starting library of biotinylated RNA:DNA substrates is prepared as in standard NAIM so that each individual substrate bears a phosphorothioate-containing nucleotide analog within the RNA strand (Step 1). The library of substrates is affixed to streptavidin-coated magnetic beads and incubated with Rho to form Rho:substrate complexes (Step 2). After elimination of unbound species from the bead slurry, single-run unwinding reactions are initiated by addition of Mg-ATP and poly[rC] trap. Bead slurry aliquots are then withdrawn at various reaction times and mixed with quench buffer (Step 3). Supernatants containing RNA species released upon Rho action are separated with a magnet from beads bearing unreacted RNA:DNA substrates (Step 4). Total RNA species present in each bead and supernatant fraction are subjected to iodine treatment (to cleave phosphorothioate linkage tags) and analyzed by sequencing PAGE (Step 5). Then, reaction progress curves are calculated for each band position of the sequencing profile from the variations in band intensities observed as a function of time for the bead and supernatant fractions. In this way, a ‘kinetic trace’ can be assigned to each species of the initial library of substrates, reflecting the kinetic effect induced by the specific nucleotide modification contained (at a given position) in the individual species (Step 6).

Bead fractionation has been used successfully to monitor Rho-induced disruption of transcription elongation complexes (29–31). To assess if helicase reaction species can be similarly separated and analyzed from bead-affixed complexes, we immobilized a variant of substrate C having a 5′-biotinylated d(T)_10_ tail (substrate C_Bt_) on streptavidin-coated magnetic beads (Figure 3). Beads were incubated with an excess of Rho before being washed with helicase buffer to remove unbound molecules. Helicase reaction was then initiated by addition of Mg-ATP. The initiation mix also contained poly[rC] in order to trap Rho molecules that are released from the beads and thus prevent their recycling onto RNA:DNA substrates (single-cycle conditions) (28,32). Supernatant and bead fractions were separated at various times from reaction aliquots and analyzed by denaturing PAGE. The fraction of duplex unwound at each sampled time was determined by comparing the RNA contents of the supernatant and bead fractions (Supplementary Figure S4, Method 1). The kinetics of duplex unwinding determined by this procedure was undistinguishable from kinetics obtained by analyzing the contents of reaction aliquots directly (Supplementary Figure S4, Method 2). This concordance indicates that bead fractionation and subsequent recovery of RNA species from the fractions are sufficiently quantitative to monitor Rho helicase reactions directly.

**Figure 4.**
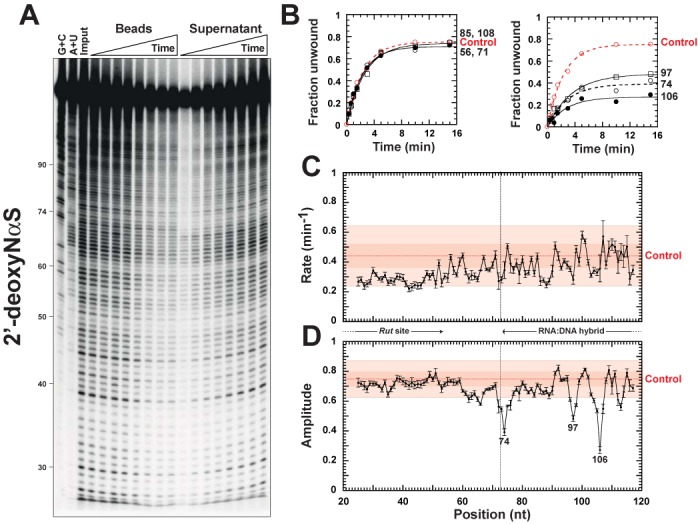
The trNAIP probing of Rho translocation/unwinding activity using 2′-deoxyNαS interference probes. **(A)** A representative PAGE gel showing sequencing band ladders obtained for the bead and supernatant fractions as a function of helicase reaction time. Gamma settings were adjusted to facilitate visualization of time-dependent band changes. **(B)** Examples of trNAIP traces obtained for RNA track positions where a 2′-deoxy NαS incorporation either has no (left graph) or a significant (right graph) kinetic effect. The ‘Control’ curve was derived from reaction aliquots that were analyzed directly, without iodine-directed sequencing (Supplementary Figure S4, Method 2), and is averaged from 12 independent experiments. The summaries of the pseudo-first order helicase rate constants and reaction amplitudes determined as a function of the position of 2′-deoxy NαS incorporation within substrate C_Bt_ are shown in panels **(C)** and **(D)**, respectively. Data points deviating by less than 1 or 2.5 SD from ‘Control’ values fall within the dark and light red boxes (68.3% and 98.8% confidence intervals), respectively.

To assess if kinetic traces attributable to individual chemical modifications of the RNA track can be generated with the above procedure, experiments were repeated with C_Bt_ substrates containing 2′-deoxy-NαS modifications at random positions of the RNA strand (Figure 3, steps 1–4). The bead and supernatant fractions of every reaction aliquot were then individually treated with iodine, to cleave phosphorothioate linkages (24), and analyzed by sequencing PAGE (Figure 3, step 5). To maximize the method throughput, all positions of 2′-deoxy-NαS modification were monitored simultaneously, which contrasts with NAIM wherein base specific subsets (A, C, G, U) are analyzed in parallel (Figure 2B). As shown in Figure 4A, this procedure resulted in sequencing band ladders whose global intensities decreased or increased as a function of reaction time for the bead or supernatant fractions, respectively. Band intensities were measured individually and used to build kinetic traces (Figure 3, step 6) corresponding to Rho-directed unwinding of RNA:DNA substrate species, each containing a 2′-deoxyNαS modification at a position located between positions 25 and 118 of the RNA track (analysis of outermost positions requires distinct experimental conditions and was not performed here). For the 94 positions analyzed (and the ‘Control’ trace), data points were best fitted with a single exponential equation (Figure 4B; Supplementary Figures S4 and S5) characteristic of a single-cycle helicase reaction obeying pseudo-first order kinetics (28,33). The invariable lack of a detectable lag in the apparition of unwound species indicates that Rho unwinds all the 2′-deoxyNαS-modified substrates in reactions that remain kinetically controlled by a single step (34). All pseudo-first order rates were found to be similar to the rate of the control reaction (Figure 4B; Supplementary Figures S5 and S6; see also below), strongly suggesting that the presence of a 2′-deoxyNαS modification in the RNA track does not change the kinetic regime and, thus, that duplex unwinding always remains rate-limited by the initial, slow activation of the Rho:substrate complex (28,33).

A majority of kinetic traces are undistinguishable (∼13%) or only slightly distinct (∼60%) from the control trace (Figure 4B and Supplementary Figure S5, left graphs). Instances where a 2′-deoxyNαS modification improves the unwinding reaction are few (∼7%) and the effects are marginal at best (Supplementary Figure S5, bottom left graph). By contrast, ∼25% of 2′-deoxyNαS modifications are detrimental to the unwinding reaction and effects are generally larger (Figure 4B and Supplementary Figure S5, right graphs). Most of these detrimental 2′-deoxyNαS modifications fall in interference regions defined by NAIM within the RNA:DNA duplex region (see below). In control trNAIP experiments performed with substrates C_Bt_ containing parental NαS analogs, we did not detect significant variations of the unwinding reaction parameters (Supplementary Figure S7). This indicates that the phosphorothioate tag moieties do not play a major role in the 2′-deoxyNαS modification effects described above, which can thus be essentially ascribed to the absence of the 2′-hydroxyl moieties. Taken together, these observations validate the trNAIP approach and confirm that the 2′-OH moieties of the RNA track are not all considered equal by the translocating Rho helicase (19).

### Absence of 2′-hydroxyls groups at specific RNA track positions destabilizes the translocating Rho complex

From the kinetic traces, we derived the amplitude and pseudo-first order rate of the single-run unwinding reaction as a function of the position of 2′-deoxyNαS modification. We observed that the 2′-deoxyNαS-dependent reaction rate does not deviate significantly from the control reaction rate (Figure 4C and Supplementary Figure S6). For ∼85% of positions, the 2′-deoxyNαS-dependent reaction amplitude also does not deviate significantly from the control reaction amplitude (Figure 4D and Supplementary Figure S6). These positions include *Rut* site positions where 2′-deoxyNαS incorporations no more affect the amplitude than the rate of the unwinding reaction (Figure 4C and D). This agrees with similar Rho affinities for RNA and DNA (25), with the capacity of D^Rut^-containing substrate B (Figure 2A) to elicit Rho activity (18), and with the lack of PBS contacts to RNA sugar moieties in Rho structures (6,7,13).

By contrast, the amplitude of the unwinding reaction decreases significantly for three downstream, narrow regions of 2′-deoxyNαS modification centered around positions 74, 97 and 106 (Figure 4D). These regions are located at the beginning and in the middle of the reporter RNA:DNA hybrid and coincide with the three clusters of significant, detrimental 2′-deoxyNαS effects identified for substrate C by NAIM (Figure 2A). Smaller, localized decreases of the 2′-deoxyNαS-dependent amplitude (Figure 4D, positions 63–66 and 112–113) may not be significant as they are not detected by global statistical analysis (Supplementary Figure S6), NAIM (Figure 2A), or trNAIP with other nucleotide analogs (see below).

A significant decrease in 2′-deoxyNαS-dependent amplitude indicates that Rho, upon encountering the nucleotide modification, becomes less able to complete duplex unwinding. The probability to undergo each elementary unwinding step (step processivity) is governed by competing dissociation and unwinding pathways and is given by the ratio of *k_U_*/(*k_U_*+*k_D_*), where *k_D_* is the rate of dissociation of the transient Rho:RNA complex and *k_U_* is the rate of the next unwinding step (34). The observed decreases in 2′-deoxyNαS-dependent amplitude can be mimicked by numerical simulation of the reaction pathway upon altering a single unwinding step, either by increasing its *k_D_* or decreasing its *k_U_* (Supplementary Table S1). Such a transient, localized destabilization effect would be consistent with SBS:RNA contacts that are continuously remodeled during translocation and could reflect an elaborate regulatory mechanism whereby Rho checks its substrate lattice before undertaking translocation/unwinding steps (see Discussion). An alternative possibility could be that Rho becomes stalled on its RNA track at the position of 2′-deoxyNαS modification. In this case, both *k_U_* and *k_D_* would be significantly decreased. Although we cannot formally exclude this scenario, we suggest that stabilization of the Rho:substrate complex (lower *k_D_*) upon depletion of a key substrate's 2′-OH group (i.e. upon reducing the potential for forming stabilizing interactions) is unlikely. This suggestion is in line with experiments comparing RNA and DNA substrates which show that 2′-OH groups are not only required for enzymatic activity but also for binding of the substrate to Rho's SBS (11,12).

Using NAIM, we observed that the sequence of the RNA:DNA duplex affects the 2′-deoxy penalty landscape of Rho-directed unwinding (19). Consistent with this observation, 2′-deoxyNαS-dependent trNAIP profiles are differing for substrates having distinct duplex sequences (Supplementary Figure S8). Although 2′-deoxyNαS-dependent rate fluctuations are not significant, variations in reaction amplitude are observed for deoxyNαS modifications located in the downstream sections of the substrates (Supplementary Figure S8). This supports that periodic destabilization of the translocating Rho complex, albeit sequence-dependent, is a general consequence of the absence of a 2′-hydroxyl moiety at specific positions of the RNA track.

### An unstable, sterically constrained translocation intermediate

**Figure 5.**
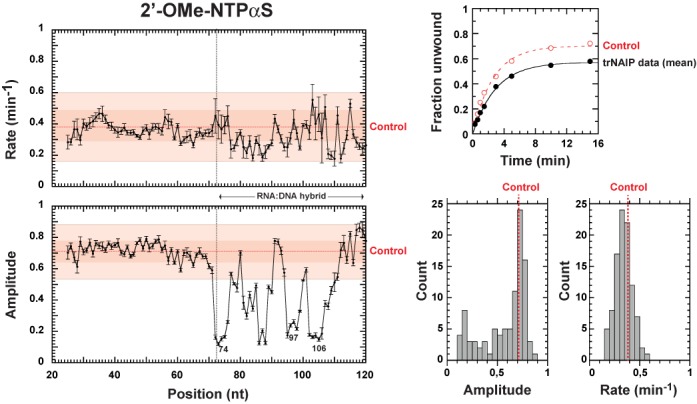
trNAIP analysis of C_Bt_ substrates containing 2′-*O*-methyl-NαS modifications. The top right graph shows pseudo-first order kinetic fits obtained with the mean values of the time points measured for ‘Control’ experiments (*n* = 12) or trNAIP data (96 positions analyzed). Histograms represent the distributions of trNAIP-derived amplitude and rate constants shown in left graphs.

Previous work excluded that the dependence of the 2′-deoxy penalty landscape on the sequence of the RNA:DNA duplex stems from differences in base pairing strengths (19). Rather, the data supported that these variations are primarily due to sequence-dependent modulation of the SBS:RNA interaction network within the central Rho channel (19). To test whether steric constraints contribute to the structural accommodation of the RNA chain within the SBS, we performed trNAIP experiments with C_Bt_ substrates containing 2′-OMe-NαS analogs. The 2′-*O*-methyl group is about twice bulkier than the 2′-hydroxyl moiety (Supplementary Table S2) and can only act as a H-bond acceptor (24). These features strongly affect the capacity of Rho to unwind the C_Bt_ substrates (Figure 5). While the 2′-OMe-NαS-dependent reaction rate is not significantly impacted, the 2′-OMe-NαS-dependent amplitude displays large decreases (up to ∼80%) in the RNA:DNA duplex region (Figure 5). Amplitude effects are significantly stronger than for 2′-deoxyNαS modifications (Figure 4D) and distributed in rather evenly spaced interference ‘valleys’ along the RNA:DNA duplex region (Figure 5). Using the same line of reasoning than for 2′-deoxyNαS effects, we conclude that the presence of a 2′-*O*-methyl group at specific positions of the RNA track strongly destabilizes the translocating Rho complex. Destabilization occurs in a pseudo-periodic pattern (Figure 5) suggesting that the complex continuously oscillates between two extreme states that considerably differ in their stability and dependence on 2′-OH group(s) and steric constraints.

**Figure 6.**
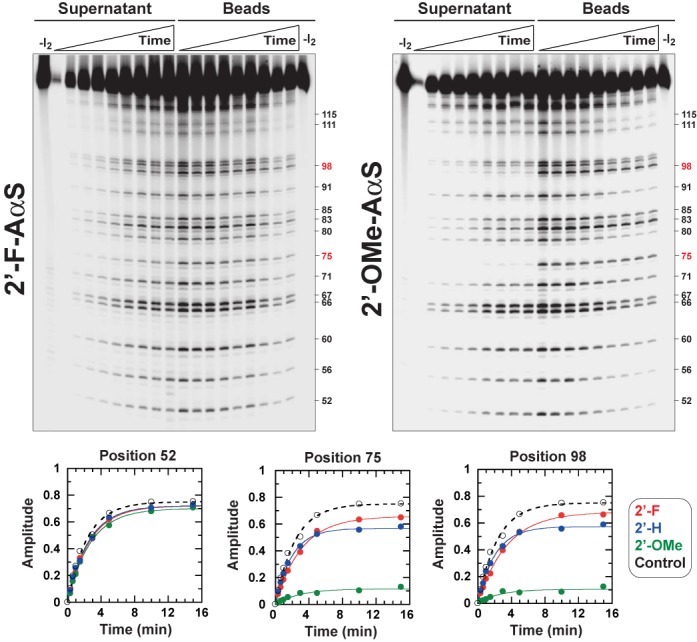
Comparison of the trNAIP effects induced by modification of substrates C_Bt_ with 2′-Fluoro-AαS or 2′-*O*-methyl-AαS analogs. Positions of most significant effects are indicated in red on the right sides of gels. Gamma settings were adjusted to facilitate visualization of time-dependent band changes. The graphs compare the kinetic effects of 2′- modifications (key is inset) for relevant positions of the substrate.

To confirm that interference enhancements elicited by 2′-*O*-methyl groups (compare Figures 4D and 5) stem from steric effects, we performed trNAIP experiments with C_Bt_ substrates containing 2′-F-AαS analogs (other 2′-F-NTPαS analogs were not commercially available). The 2′-F atom is slightly smaller than the 2′-hydroxyl moiety (Supplementary Table S2) but is a strong H-bond acceptor (24). Incorporation of 2′-F-AαS analogs in the RNA track affects Rho-directed unwinding at a couple of positions within the duplex region (Figure 6). Effects are comparable to those triggered by 2′-deoxy modifications at the same positions but are much weaker than those triggered by 2′-*O*-methyl groups (Figure 6; also note the remarkably similar ‘chemo-kinetic’ graphs for positions 75 and 98, consistent with Rho going through the same 2′-OH-dependent process/state at periodic intervals). This trend of effects was confirmed in NAIM experiments with substrates A, B and C (Supplementary Figure S9). These data support that steric bulkiness largely contributes to the strong 2′-*O*-methyl interference effects observed by trNAIP (Figure 5). Yet, that both small 2′-F and large 2′-*O*-methyl moieties elicit interference effects also suggests that strict H-bond acceptors are not adequate substitutes for the key 2′-OH groups that stabilize the translocation complex at periodic intervals. Rather, stabilization appears to rely on the development of a hydrogen bond between a 2′-hydroxyl H atom and an acceptor SBS residue. Interestingly, the H-bond acceptor carbonyl groups of Val284 residues engage 2′-hydroxyl RNA groups in the asymmetric ring structure of Rho (14). Asp322 side-chains appear to have similar capacity in the ‘trimer of dimers’ structure (13). However, Asp322 residues are poor SBS candidates because they can be mutated to alanine without deleterious consequences for the enzymatic activities of Rho (18).

## DISCUSSION

Transcription termination factor Rho predominates as a ring-shaped homo-hexamer under physiological conditions (25). This functional Rho hexamer is a tightly-regulated RNA translocase. Regulation starts upon recognition of a transcript *Rut* site by the crown-like PBS of the Rho hexamer (‘anchoring’ step). This step is a key event initiating the rate-limiting, multistep formation of a catalytically active Rho:RNA complex (5,25). Biochemical (15) and single-molecule ((16); our unpublished observations) experiments argue that PBS contacts to the *Rut* site persist during RNA translocation. This ‘tethered tracking’ mechanism is supported by the differences in 2′-deoxyNαS penalty landscapes observed for RNA:DNA substrates that vary only in their upstream, single-stranded RNA ‘anchoring’ sections (Figure 2). These differences also suggest that the persistent PBS:RNA interaction is not neutral but rather modulates ‘at distance’ the translocation and unwinding behavior of Rho. This may be due to allosteric coupling between the PBS and SBS (18,35) and/or to structural constraints that develop within the RNA loop formed between the PBS and SBS during translocation. The persistent PBS:RNA interaction may also limit ‘off-pathway’ states such as ones resulting from transient openings of the Rho ring. Stabilization of active Rho isoform(s) by NA circling on the PBS is corroborated by poly[dC]-coupled ATPase experiments whereby poly[dC] (which only binds to the PBS) strongly stimulates ATP hydrolysis induced by short, SBS-bound oligoribonucleotides (10,36).

Several lines of evidence support that the conversion of the initial, split-open Rho hexamer into a closed, catalytically competent configuration is governed by Rho:RNA contacts extending from the PBS to the SBS (3–5,13). This activation process is strictly specific for RNA (12,19) and most likely involves significant rearrangement of SBS residues (Supplementary Figure S10). This model predicts that features/events affecting the strength or extent of the SBS:RNA interaction network will shift the balance toward the reverse pathway ([closed → open] ring conversion), thereby triggering regulatory checkpoints during translocation (see below). Existence of unstable translocation intermediates is supported by the moderate unwinding processivity (60–80 base pairs) of Rho (28,33). A higher processivity may have been expected for a ring-translocase encircling its NA substrate (1–3).

Congruent with NAIM data (Figure 2), our trNAIP analysis shows that alterations of 2′-hydroxyl groups at discrete positions of the RNA track affect Rho translocation/unwinding activity. This is evidenced by significant, position-specific decreases in the amplitude of the unwinding reaction (Figures 4–6) which, under the single-run helicase conditions of the trNAIP assay, indicates that the 2′-modifications favor dissociation of the Rho:RNA complex. A general design principle for processive motors considers that any on-pathway state that is vulnerable to dissociation requires a mechanism for rapid exit from this state into a more tightly-bound configuration (37,38). Thus, a plausible explanation of our data is that 2′-modifications prevent fast escape of the Rho motor from a vulnerable state forming periodically (every ∼7-nt) (19) during translocation (Supplementary Table S1). These 2′-modifications may also (or alternatively) render the Rho:RNA complex more vulnerable in this state (i.e. loss of SBS contact to a 2′-OH moiety increases its dissociation rate). In any case, the SBS:RNA interaction network is weakened within the vulnerable translocation intermediate to the extent that the absence of a single 2′-OH interacting moiety (Figure 4), or its replacement by a H-bond acceptor group (Figures 5 and 6; Supplementary Figure S9), is sufficient to promote dissociation of the Rho:RNA complex. Moreover, both steric (2′-*O*-methyl) bulk (Figure 5) and RNA sequence (Supplementary Figure S8) (19) effects are consistent with structural stress in the vulnerable intermediate (due, for instance, to tight constraints on the RNA spiral staircase within the SBS; (14)). Sequence effects may also reflect a transient SBS interaction network including contacts to RNA base moieties as observed in the ‘trimer of dimers’ (13) and, to a lesser extent, the ‘asymmetric’ ring (14) structures.

**Figure 7.**
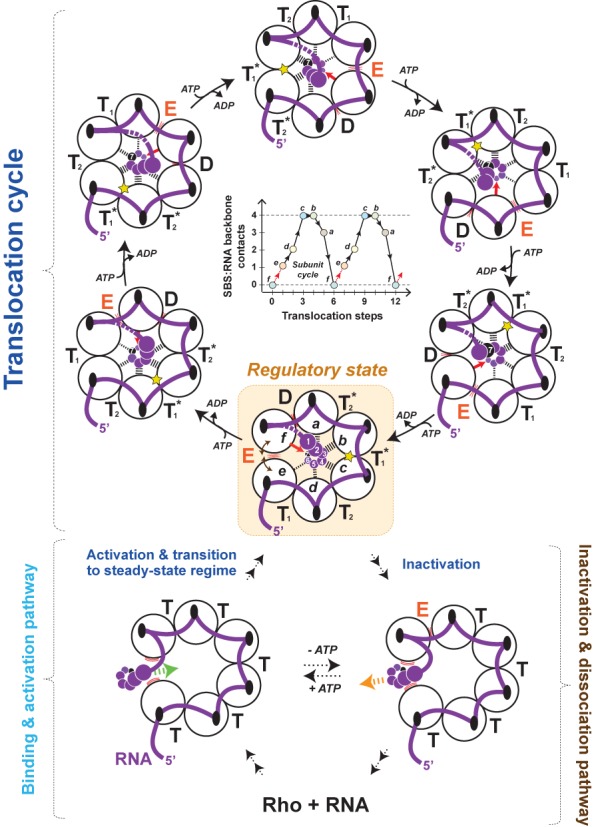
A model for RNA translocation by the Rho helicase. Black dashed lines depict contacts between RNA sugar or phosphate moieties and putative SBS residues in the Q and R loops of Rho subunits as is observed in the ‘asymmetric’ ring structure (14). The red arrows symbolize the first SBS:RNA interaction to be formed between the 2′-OH group (H-bond donor) of the incoming 3′RNA nucleotide and the Val284 carbonyl group (H-bond acceptor) of the engaging subunit (see also Supplementary Figure S11). D, E, T and T^*^ represent the distinct nucleotide occupancy states formed during the ATPase cycle (14). The Rho hexamer is shown from the top with black ovals representing PBS clefts on individual subunits. Formation of a productive Rho:RNA complex initially requires binding and circulation of the RNA chain (thick purple line) on the crown-like PBS of a split-open Rho ring (bottom left). Entry of a downstream section of the RNA chain (nucleotides depicted as spiraling purple and black circles) into the ring channel and contacts with a subset of SBS residues trigger ring closure and formation of a catalytically competent Rho:RNA complex. Rho adopts the ‘asymmetric’ ring configuration to sustain the catalytic steady-state regime but PBS:RNA contacts are not released. The persistent PBS:RNA interaction contributes to the ring asymmetry throughout the translocation cycle (dotted purple segment depicts the RNA loop that develops between the PBS and SBS) with one ‘weak’ interface that is not PBS-bridged by RNA. This interface is weakest every seventh nucleotide when its *f*-state subunit needs to engage a new RNA nucleotide and when a new ATP cofactor has to be loaded (‘regulatory’ state). At this stage, the strongest interface (yellow star) is directly opposite the ‘weak’ interface within the Rho ring. Escape from the ‘regulatory’ state critically depends on engagement of the 2′-OH group of the incoming 3′-RNA nucleotide by the V284 residue of the *f*-state subunit (see Supplementary Figure S11). If the 2′-OH group is absent or not readily accessible, ‘breathing’ (symbolized by brown double-headed arrows) of the ‘weak’ interface allows reversion toward a split-open, inactive state with RNA outside the Rho ring. RNA exit at other interfaces is less probable due to the intersubunit stabilization and physical barriers brought by RNA segments spanning PBS subsites.

Taking these observations into account, we propose a new model for Rho translocation that includes salient features from the crystal structures of Rho (7,9,13,14). We assume that formation of a catalytically active Rho:RNA complex (Figure 7, activation pathway) involves the RNA-dependent [open → closed] ring conversion described above. Significant structural changes are expected during this process (Supplementary Figure S10). Moreover, upon RNA entry into the ring, transient RNA contacts may form transiently at the subunit interface in a way reminiscent to that seen in the ‘trimer of dimers’ structure (Figure 1B) (13). Importantly, the persistence of PBS:RNA contacts implies that the subunit interface used for RNA entry will bear less ‘cohesive’ potential than the other interfaces during the subsequent translocation/unwinding pathway. This is indeed the only subunit interface that is not fastened by RNA bridging its PBS clefts (Figure 7). This feature has been overlooked in previous ‘tethered tracking’ models of Rho (15,16). We suggest that this subunit interface (hereafter, called ‘weak’ interface) is essentially devoid of ATP cofactor until Rho reaches a steady-state catalytic regime. This proposal is consistent with equilibrium binding measurements showing that, in presence of poly[rC], only five ADP-BeF_3_ analogs bind per Rho hexamer at saturating nucleotide concentrations (39).

Once RNA is in the central channel, Rho should adopt a conformation suitable for RNA translocation. We propose that this conformation is akin to the ‘asymmetric ring’ structure (14) but with RNA remaining affixed to the PBS (Figure 7). Strictly speaking, the ‘asymmetric ring’ configuration with subunit interfaces in four distinct ATPase cycle states (T, T*, D and E states; Figures 1 and 7) most likely represents Rho in a steady-state catalytic regime. Rho may thus first hydrolyze several ATP molecules (without translocating RNA) to reach this steady-state configuration. This would be consistent with transient kinetics evidence for an intermediate ‘activation’ complex that is able to hydrolyze three ATP molecules in a burst but is not yet capable to catalyze ATPase turnover (40).

Once the steady-state ‘asymmetric ring’ configuration is acquired (Figure 7, regulatory state), RNA is threaded through the Rho ring in a succession of 1-nt steps according to ‘escort’ models based on staircase arrangements of NA-subunit contacts (14,17). Each incoming 3′-RNA nucleotide is recruited by a new Rho subunit (Figure 7, red arrows) evolving from the transient *f* conformation (as defined in (14)) (Supplementary Figure S11A). The RNA chain is rotated by 360° around the ring axis every seventh nucleotide when the first subunit to finish its nucleotide escort engages a new nucleotide (Figure 7) (14). After one cycle of ATP hydrolysis around the Rho ring, the subunit that needs to transition from the top to the bottom of the ring in order to escort a new nucleotide (14) is thus the subunit in the *f* conformation at the ‘weak’ interface (Figure 7, regulatory state). Repositioning of the subunit will bring the backbone carbonyl group (H-bond acceptor) of its Val284 residue in the proximity of the 2′-hydroxyl group (H-bond donor) of the incoming 3′-RNA nucleotide (Supplementary Figure S11A and B). Importantly, the interaction between these two functional groups will be the first contact formed in the SBS:RNA interaction network that develops as the subunit escorts the nucleotide through the Rho ring (Supplementary Figure 11A) (14). Overall, three translocation steps will be required for the subunit to switch from its RNA-free conformation *f* to its tightest RNA-bound conformation *c* (Figure 7, inset) (14).

The escort model of Rho suggests that a subunit interface is most likely to break when it goes through the nucleotide-exchange state E (Figure 7). This interface is the least stabilized by intersubunit and SBS:RNA contacts (14) and risks further destabilization by the exchange of nucleotide cofactor (we expect nucleotide-free states to be less stable than ATP/ADP-bound states; see (41)) and the top to bottom repositioning of its *f*-state subunit (Supplementary Figure S11A and B). We propose that, without RNA bridging its PBS subsites, the ‘weak’ interface is even more susceptible to breakage when it goes through the E state. This premise defines a regulatory state in the translocation cycle of Rho every time the ‘weak’ interface goes through the E state (Figure 7, every seventh RNA nucleotide). Escape from the regulatory state will then depend critically on the capacity of the *f*-state subunit to engage the incoming 3′-RNA nucleotide through formation of a SBS (i.e. Val284 carbonyl) contact with its 2′-OH group (Figure 7 and Supplementary Figure S11A and B).

Accommodation/rearrangement of key interacting residues within the structurally congested Rho ring (Supplementary Figure S11C) should be strongly sensitive to RNA bulk, thereby explaining sequence (Supplementary Figure 8) (19) and 2′*O*-Methyl group (Figure 5) interference effects. The fate of Rho in the regulatory state may also depend on the likelihood of forming interaction(s) between the *f*-state subunit and nucleotide base moieties (Supplementary Figure S11D). Although other interfaces entering the nucleotide-exchange state E may be susceptible to some extent to the same factors, RNA bridging their PBS clefts should restrict the large structural changes required to inactivate Rho irreversibly (Figure 7). In this respect, one should note that PBS:RNA contacts can also form outside the crystallography detected PBS clefts (26,42), thereby further securing RNA-bridged interfaces. Finally, interfaces fastened by PBS:RNA contacts may bind ATP faster or more easily, also contributing to stabilize the Rho hexamer and its interaction with RNA (41). All these features should be largely independent of the direction of RNA circling on the PBS which remains debated (9).

Our model is reminiscent of the burst (translocation) and dwell (regulation) operating mechanism proposed recently for the ring-shaped DNA translocase ϕ29 (43). In both cases, the translocation cycle is regulated by a ‘special’ subunit contacting the NA lattice. In the case of the ϕ29 translocase, however, the interaction is proposed to facilitate the release of nucleotide products and reloading of ATPase pockets which had all been fired during the burst phase (43). In the case of Rho, ATP hydrolysis, product release and ATP reloading occur at every step of the translocation cycle (Figure 7) (14). Moreover, the ‘special’ subunit is defined in Rho by the asymmetric circulation of RNA on the crown-like PBS (Figure 7). The contribution of the ‘special’ subunit to the functional regulation of Rho is hard to evaluate because key regulatory determinants such as terminator sequences or protein cofactors (4,44,45) remain insufficiently characterized. We can only speculate that the structure/bulk of specific RNA sequences or interactions with cofactors (for instance, ones capable to stabilize the weak interface) may modulate the outcome from the regulatory state (Figure 7) and, thus, the processivity of the Rho translocase. Whether the ‘special’ subunit in Rho (or ϕ29) responds to specific sequences during translocation and whether cofactors bring similar ‘regulatory’ asymmetries in translocase rings devoid of ‘loader’ domains thus remain open, intriguing questions.

In sum, our data provide key insights into the translocation and regulatory mechanisms of Rho, an important specimen of ring-shaped molecular motor. Moreover, the versatility and depth of the trNAIP methodology should make it particularly useful for the mechanistic analysis of other RNA-based systems. The key step in trNAIP is time-dependent affinity bead partitioning of reactive species (Figure 3). This procedure should be easily adaptable to many RNA-based systems in ways similar to functional SELEX assays (46). Even systems with very fast kinetics might be analyzed by trNAIP with the advent of submicrometer, low density affinity beads usable in quench flow instruments. Moreover, trNAIP is not limited to combinatorial ‘chemo-kinetic’ profiling of RNA backbone groups and can be extended to include the many nucleotide base analogs that have been developed for NAIM (24). Preliminary experiments with model ribozymes suggest that trNAIP performed with an extended set of nucleotide analogs could be very effective to analyze the functional roles and dynamics of non-canonical RNA interaction networks (our unpublished observations), thereby ideally complementing classical RNA structure probing tools.

## SUPPLEMENTARY DATA

Supplementary Data are available at NAR Online.

SUPPLEMENTARY DATA
