# Supplementary material for: The RNA-mediated, asymmetric ring regulatory mechanism of the transcription termination Rho helicase decrypted by time-resolved Nucleotide Analog Interference Probing (trNAIP)
Source: Nucleic Acids Res. 2014 Jul 12;42(14):9270–84. doi: 10.1093/nar/gku595 (PMC4132721; doi:10.1093/nar/gku595)
Supplement: SUPPLEMENTARY DATA [file supp_42_14_9270__index.html]

The RNA-mediated, asymmetric ring regulatory mechanism of the transcription termination Rho helicase decrypted by time-resolved Nucleotide Analog Interference Probing (trNAIP) — The RNA-mediated, asymmetric ring regulatory mechanism of the transcription termination Rho helicase decrypted by time-resolved Nucleotide Analog Interference Probing (trNAIP) — SUPPLEMENTARY DATA 

# The RNA-mediated, asymmetric ring regulatory mechanism of the transcription termination Rho helicase decrypted by time-resolved Nucleotide Analog Interference Probing (trNAIP)

## SUPPLEMENTARY DATA

**Files in this Data Supplement:**

- SUPPLEMENTARY DATA
