# Supplementary material for: The RNA-mediated, asymmetric ring regulatory mechanism of the transcription termination Rho helicase decrypted by time-resolved Nucleotide Analog Interference Probing (trNAIP)
Source: Nucleic Acids Res. 2014 Jul 12;42(14):9270–84. doi: 10.1093/nar/gku595 (PMC4132721; doi:10.1093/nar/gku595)
Supplement: SUPPLEMENTARY DATA [file supp_gku595_nar-01395-f-2014-File009.pdf]

The RNA-mediated, asymmetric ring regulatory mechanism of the transcription termination Rho helicase decrypted by time-resolved Nucleotide Analog Interference Probing (trNAIP)

Emilie Soares, Annie Schwartz, Marcello Nollmann, Emmanuel Margeat, and Marc Boudvillain

(Supplementary data)

Supplementary table S1 : Reaction parameters obtained upon kinetic simulation of the unwinding reaction[a]

Unwinding scheme

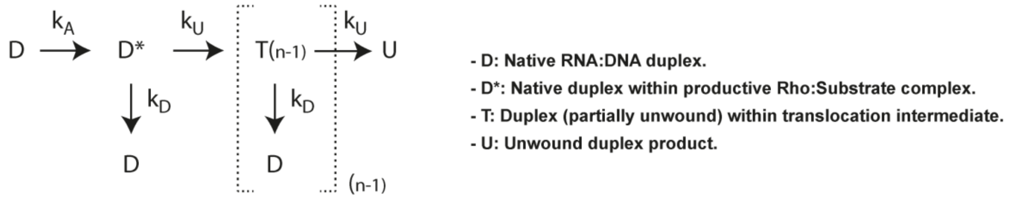

**Unwinding steps (*n*)**	**Activation**	**Normal unwinding step(s)**		**Altered (dNαS-dependent) unwinding step**[d]	
				**Case 1**[e] *(unchanged k_D)*	**Case 2**[e] *(unchanged k_U)*
	**k_A**	**k_U**[b]	**k_D**	**k_U***	**k_D***
**1**	0.35	60[c]	22.2[c]	8.4	160
**2**	0.36	120	20.4	9.5	257
⋮					
**5**	0.37	300	19.5	10.7	549
⋮					

[a]Rates, in min^{-1}, were determined with the Tenua software, assuming the simple mechanism depicted in the top scheme (one activation step followed by *n* identical unwinding steps; competitive dissociation of the Rho:Substrate complex before each unwinding step) and that no more than one of the unwinding steps is altered by the presence of a 2'-deoxyNαS modification in the RNA track. Results for several, arbitrarily-chosen *n* values are shown, as the exact number of steps is unknown.

[b]The values of k_U were arbitrarily defined assuming that, once active, Rho requires ~1 s to unwind the RNA:DNA hydrid (which is consistent with a translocation rate of ~50 nt/s measured in single-molecule experiments [our unpublished observations]).

[c]Values are for the control reaction performed with unmodified substrate C_{Bt} (Figure 4B).

[d]Presence of a 2'-deoxyNαS modification at a critical RNA position decreases the processivity of the next unwinding step, either by reducing the unwinding rate (k_U* being the minimal value) or by increasing the dissociation rate (k_D* being the maximal value).

[e]The k_U* and k_D* values were determined by assuming that the 2'-deoxy modification affects only one of both competing pathways. Values shown in the table are for the most detrimental 2'-deoxy modification of substrate C_{Bt} (position 106).

Supplementary table S2 : Van der Waals (VdW) volumes of selected functional groups[a]

**Moiety**	-H	-OH	-F	$-OCH_3$
**VdW volume (Å^3)**	7.24	16.03	13.31	33.33

[a]Volumes were estimated from atomic VdW volumes as described (47).

47. Zhao, Y.H., Abraham, M.H. and Zissimos, A.M. (2003) Fast calculation of van der Waals volume as a sum of atomic and bond contributions and its application to drug compounds. The Journal of organic chemistry, 68, 7368-7373.

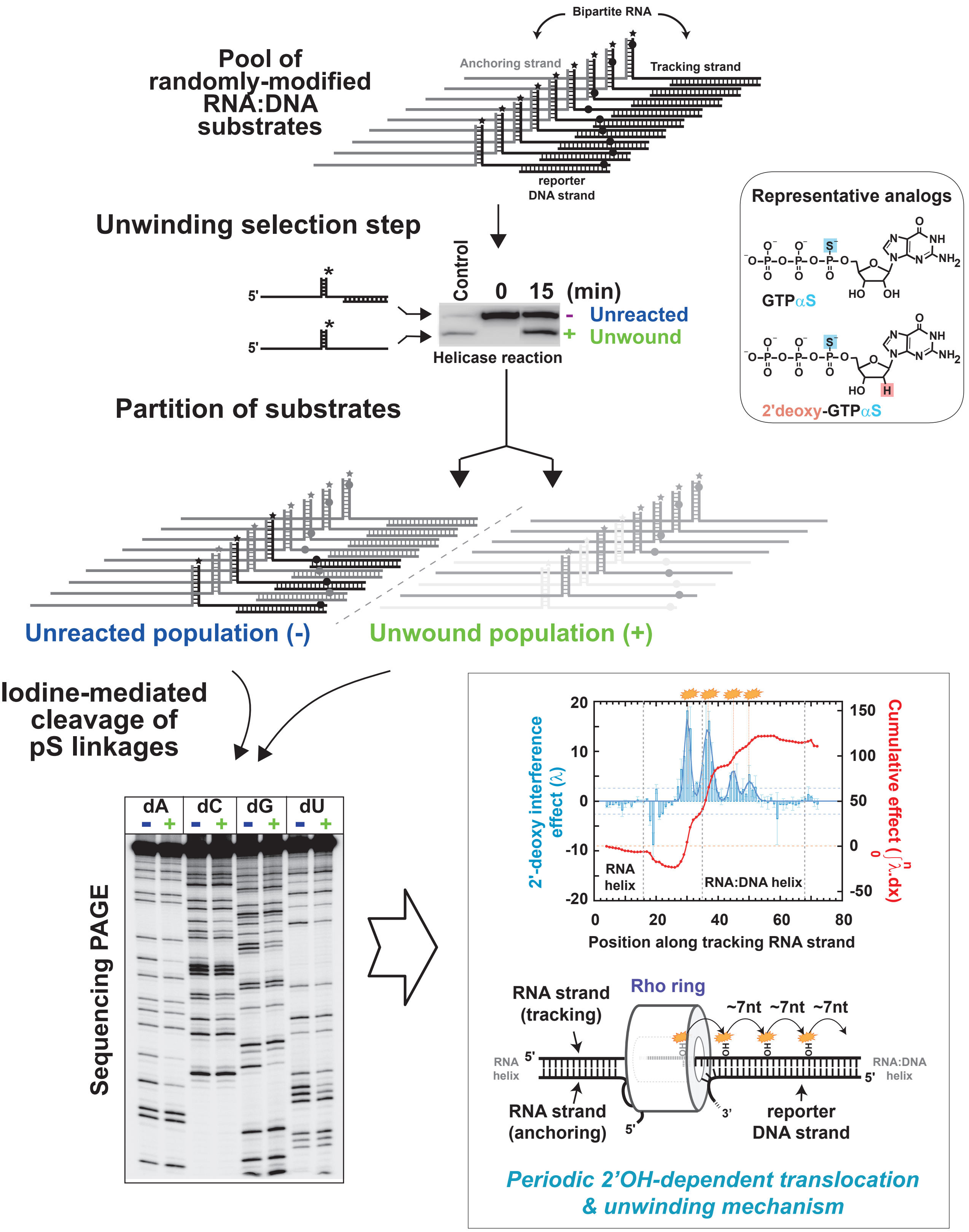

Supplementary figure S1: NAIM assay implemented for the Rho helicase.
Alpha-phosphorothioate nucleotide analogs (e.g., 2'-deoxyNαS analogs) are randomly incorporated into RNA by transcription so that each molecule of the transcribed pool contains a chemical probe of interest (e.g., a 2'-H moiety) at a distinct position. This RNA pool is used to prepare a library of chemically modified RNA:DNA substrates (the case of '3-piece' substrates, such as substrates A and B in Fig. 2A, is illustrated in the diagram) which are subjected to Rho unwinding activity. This permits the partition of the initial library of substrates into populations of functionally active (containing 'silent' or favorable RNA modifications) and defective (containing detrimental RNA modifications) species. Following iodine-directed cleavage of the phosphorothioate linkages, comparative PAGE analysis of both populations reveals the positions of chemical moieties (e.g., 2'-hydroxyl moieties) along the RNA chain that are important for translocation/unwinding by Rho. Positions of analogs in the 'tracking' RNA strand are symbolized by black/gray circles. Black/gray stars depict ^{32}P-labels. The graph shows periodic distribution of 2'-deoxyNαS interference effects representative of the periodic, 2-hydroxyl-dependent stepping of Rho along its RNA track (19). Note that graph data are not normalized for the small interference effects induced by the phosphorothioate tags used to mark the positions of the nucleotide analogs within RNA (22).

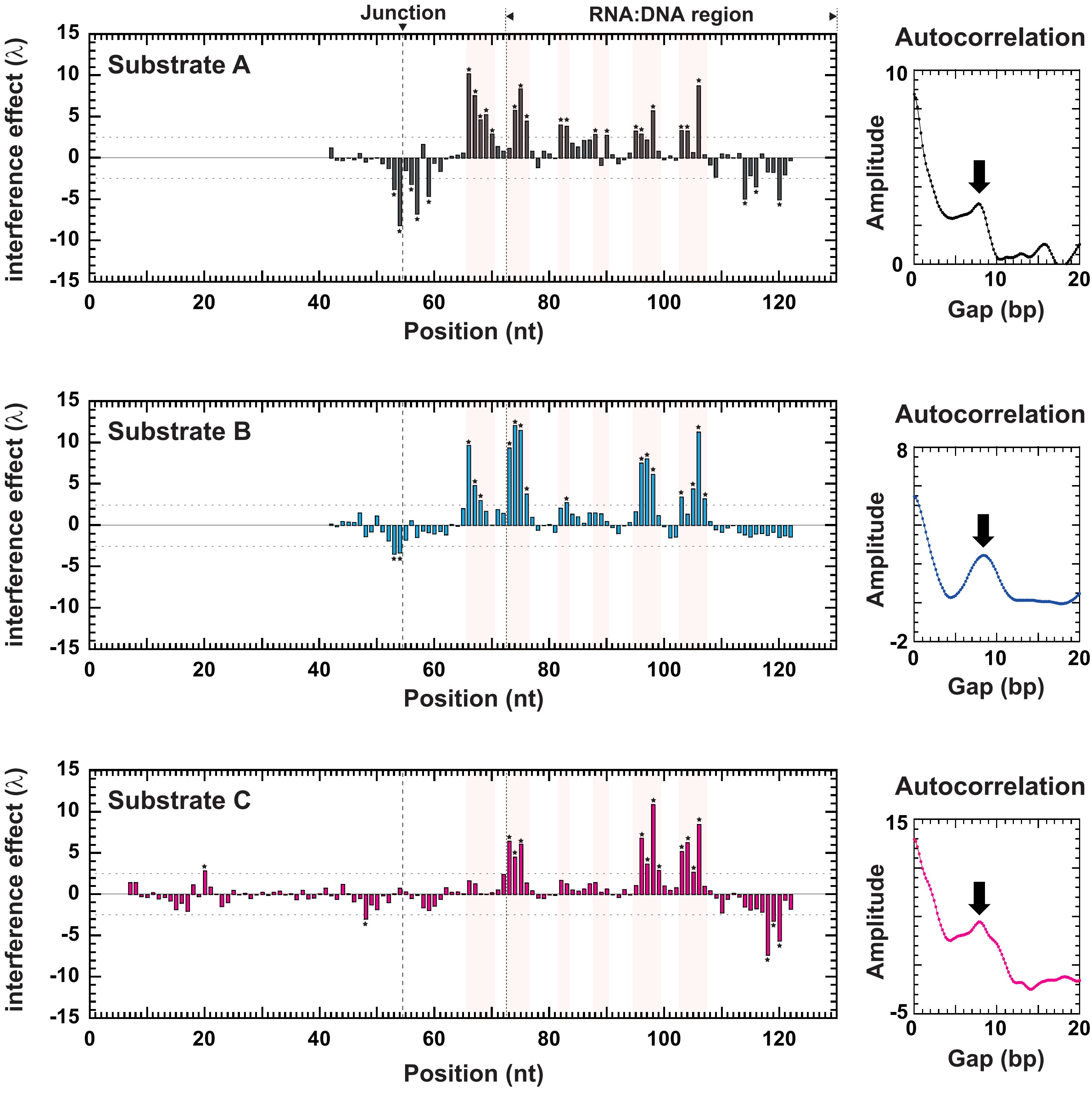

Supplementary figure S2: Summary of 2'-deoxy-NαS interference effects detected by NAIM.
The helicase activity of Rho was probed by standard NAIM of the RNA:DNA substrates shown in Figure 2A. Interference effects were normalized as described in ref. (19). Stars identify the positions of significant 2'-deoxy-NαS effects (98.8% confidence thresholds, $\lambda = \pm 2.5$; see also Fig. 2A). To facilitate comparisons, nucleotide numbering of substrate C is used in all histograms (e.g., position 60 in histograms refers to A60 in substrate C and to A22 in substrates A and B). Autocorrelation analyses were performed as described in ref. (19). Secondary autocorrelation maxima are identified by black arrows.

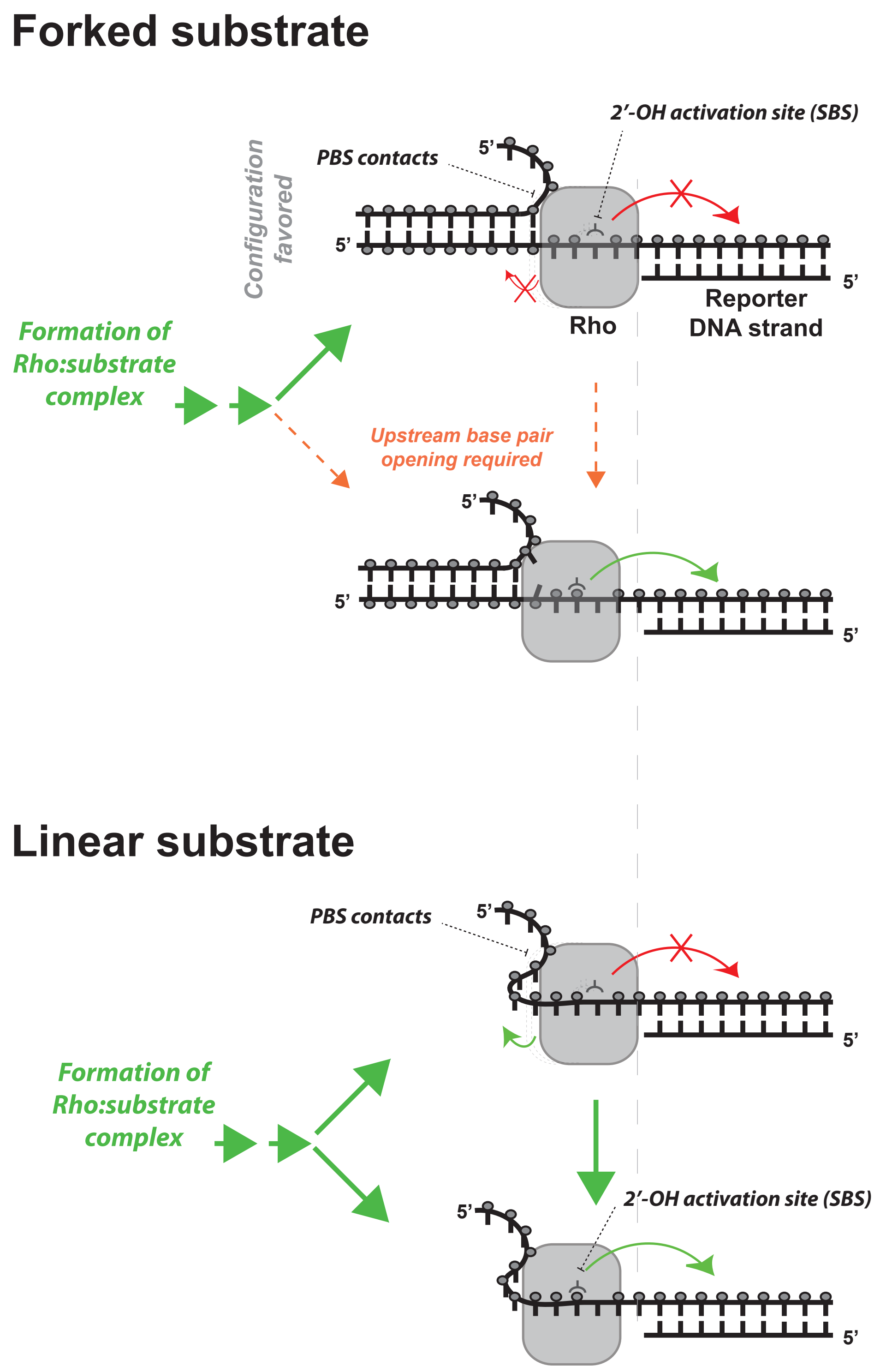

Supplementary figure S3: Structural constraints within the starting Rho:substrate complexes.
The diagram shows how the architecture of 3-piece, forked RNA:DNA constructs (top) constrains the configuration of 'starting' Rho:substrate complexes as compared to linear constructs (bottom). Grey circles depict 2'-OH moieties. The absence of a 2'-OH moiety properly positioned within the active site of Rho destabilizes the Rho:RNA complex and/or limits its capacity to translocate and unwind the reporter RNA:DNA helix. Thermal sliding of Rho along the RNA track to acquire an 'in register' 2'-OH configuration is easier for linear substrates devoid of upstream duplex structure. Direct acquisition of the productive, 'in register' 2'-OH configuration (activation pathway) should also be easier for linear substrates. These processes may occur during preincubation of Rho with the RNA:DNA substrates, even before the unwinding reaction is initiated by addition of Mg-ATP. Note that backward reactivation of Rho later on -i.e., once Rho has started unwinding the reporter RNA:DNA helix- should be equally costly for linear and forked substrates due to concomitant reannealing of the reporter helix.

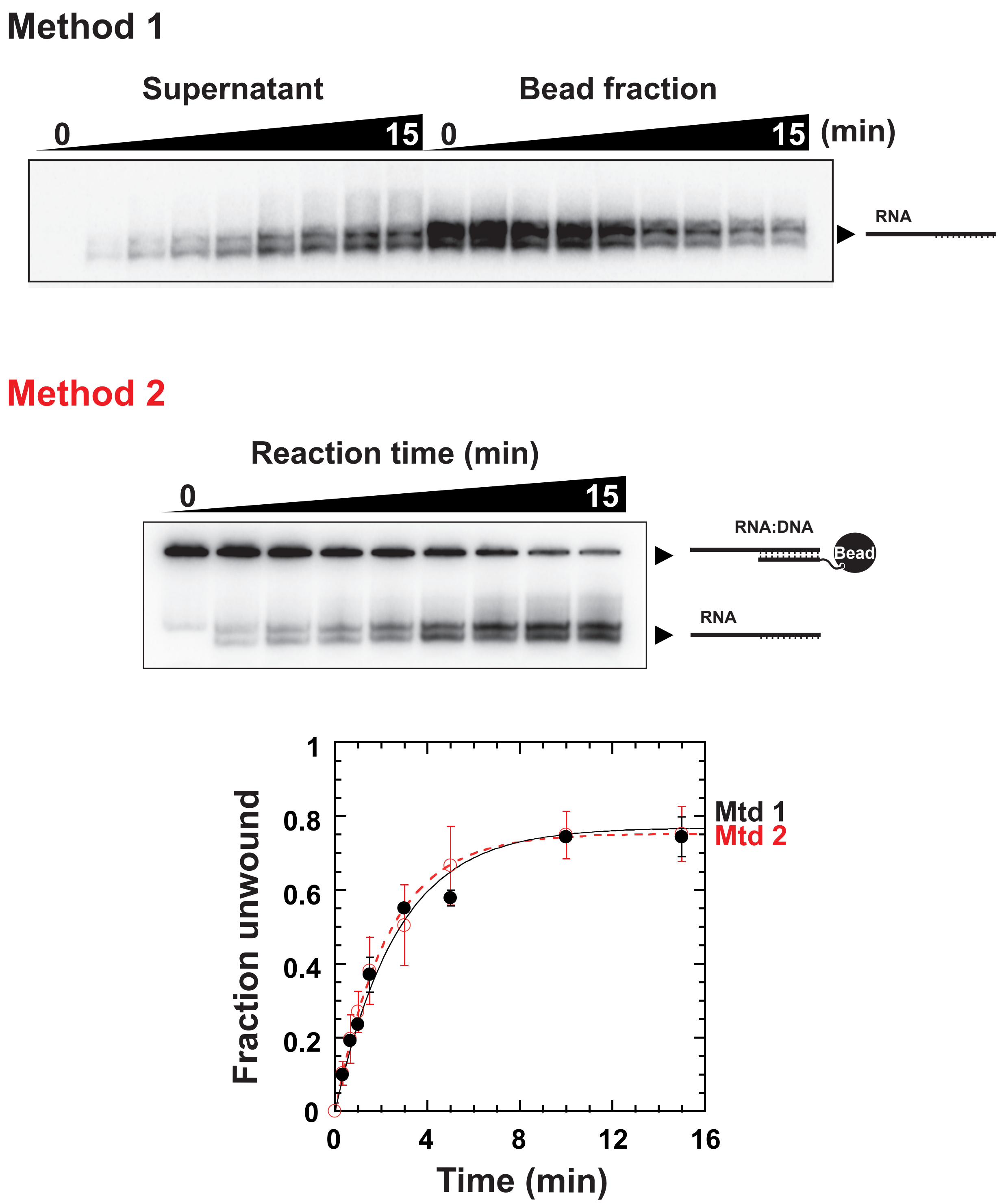

Supplementary figure S4: Rho-directed unwinding of bead-affixed RNA:DNA substrates.
Helicase experiments were performed with biotinylated substrates C immobilized on streptavidin-coated beads. Reaction aliquots were first fractionated into supernatant and bead fractions (**Method 1**; filled black circles) or loaded directly on a 8% polyacrylamide gel containing 0.5% SDS (**Method 2**; open red circles). In method 1, samples were heat-denaturated (and beads removed) under the conditions used for trNAIP (treatment with iodine excepted) before being loaded on the gel. The doublet of RNA bands is due to the presence of the oligo trap that pairs with the RNA released from the beads and prevents reannealing of the original RNA:DNA duplexes. Reactions contained ~3 nM bead-immobilized Rho:substrate complexes and were initiated with 1 mM ATP and 3 μM poly[rC] (single-run conditions). Reaction amplitudes are 0.77±0.02 and 0.75±0.05 and rates are (0.37±0.02)/min and (0.44±0.08)/min for methods 1 and 2, respectively.

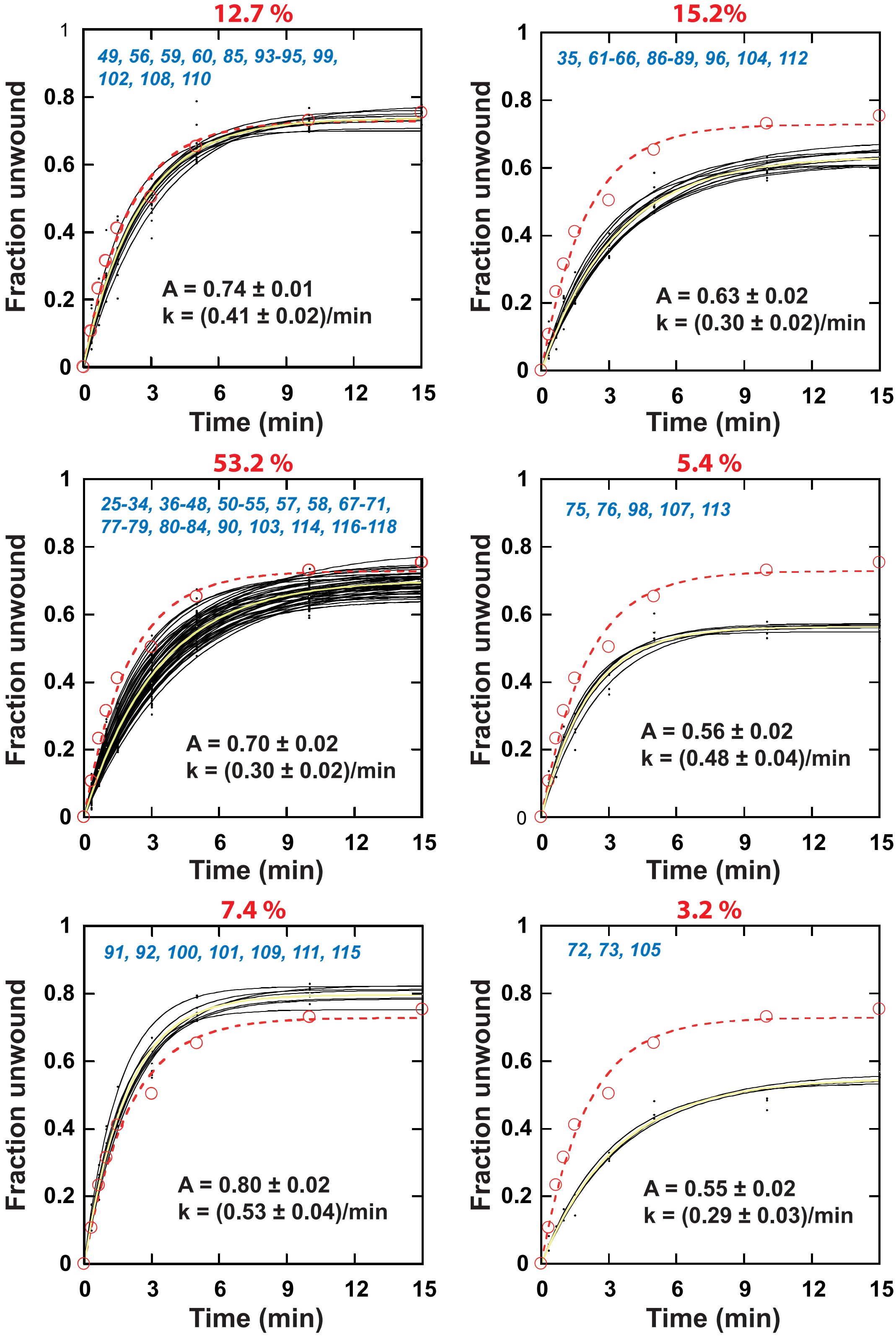

Supplementary figure S5: Kinetic traces derived from trNAIP.
Black curves represent the kinetic traces derived from trNAIP analysis of substrates C containing a 2’-deoxy-NαS modification. Kinetic traces were grouped by similarity and are shown together with a mean trace in each case (yellow curves).The positions of the 2’-deoxy-NαS modifications analyzed in each graph are indicated in blue. Amplitude (A) and rate (k) parameters shown in graphs are for the mean traces. The control curve (in red) is the same as in Figure S4 , method 1 (A = 0.75 ± 0.05 and k = [0.44 ± 0.08] min^{-1}). Note that kinetic traces for interfering 2’-deoxyNαS modifications at positions 74, 97, and 106 are not shown here but in Figure 4B. Percentages of kinetics traces in each group are shown in red above graphs.

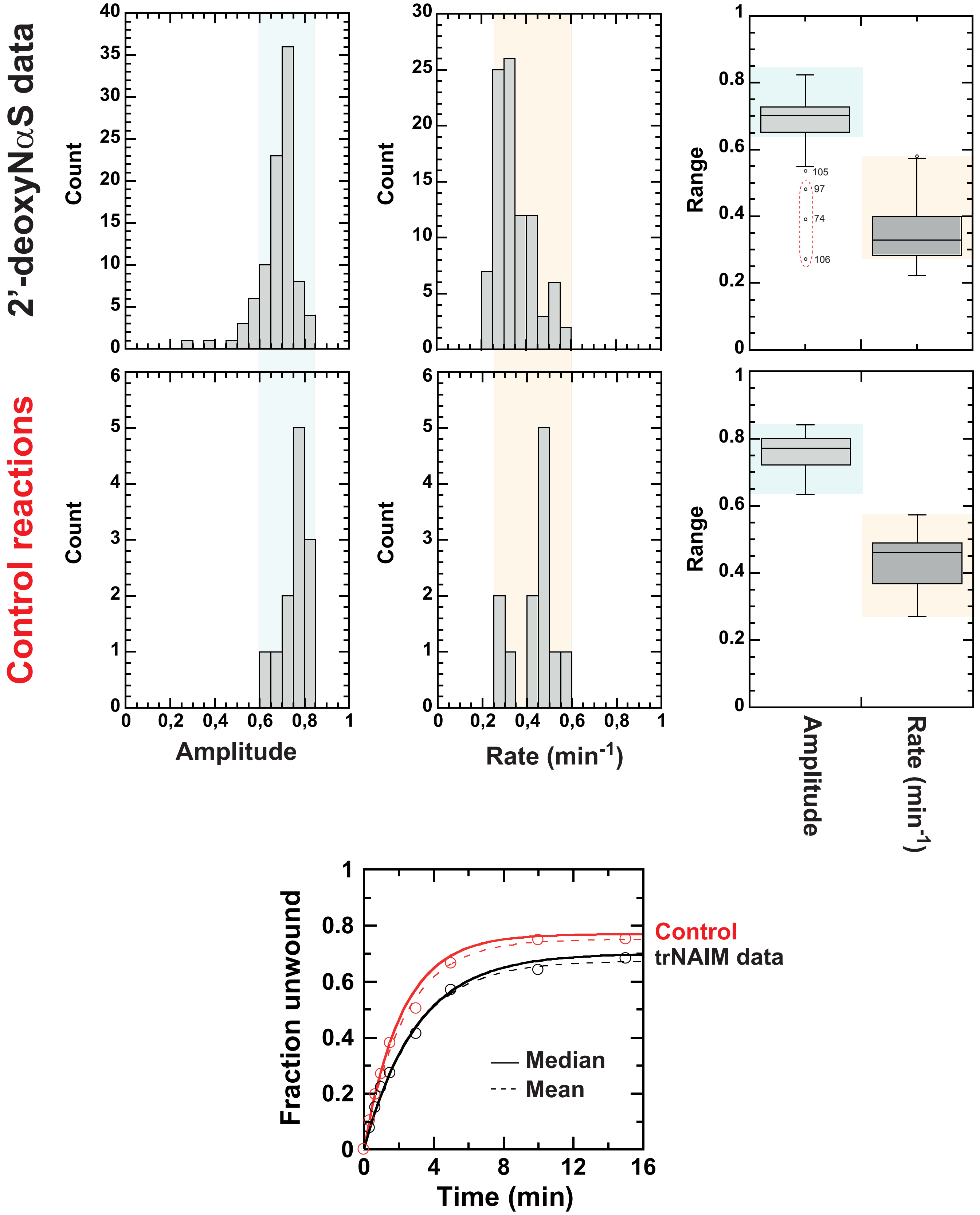

Supplementary figure S6: Statistical overview of trNAIP data.
The analysis corresponds to the trNAIP data obtained with 2'-deoxyNαS-modified substrates C (12 independent experiments) as shown in Figure S5. Boxplots show the median (inner line), interquartile range IQR (25th to 75th percentage; box), acceptable range (whiskers), and outliers (open circles). The acceptable range is delineated by the lowest and highest data points that remain within a distance of 1.5 x IQR from the box edges, as implemented in Kaleidagraph software. Light blue and yellow zones respectively include all amplitude and rate values measured for control reactions (Method 2 in Figure S4). The lower graph shows best unwinding kinetic fits obtained with the mean (circles and dotted lines) or median (solid lines) values of the time points measured for control experiments (n = 12) or the 94 positions analyzed by trNAIP.

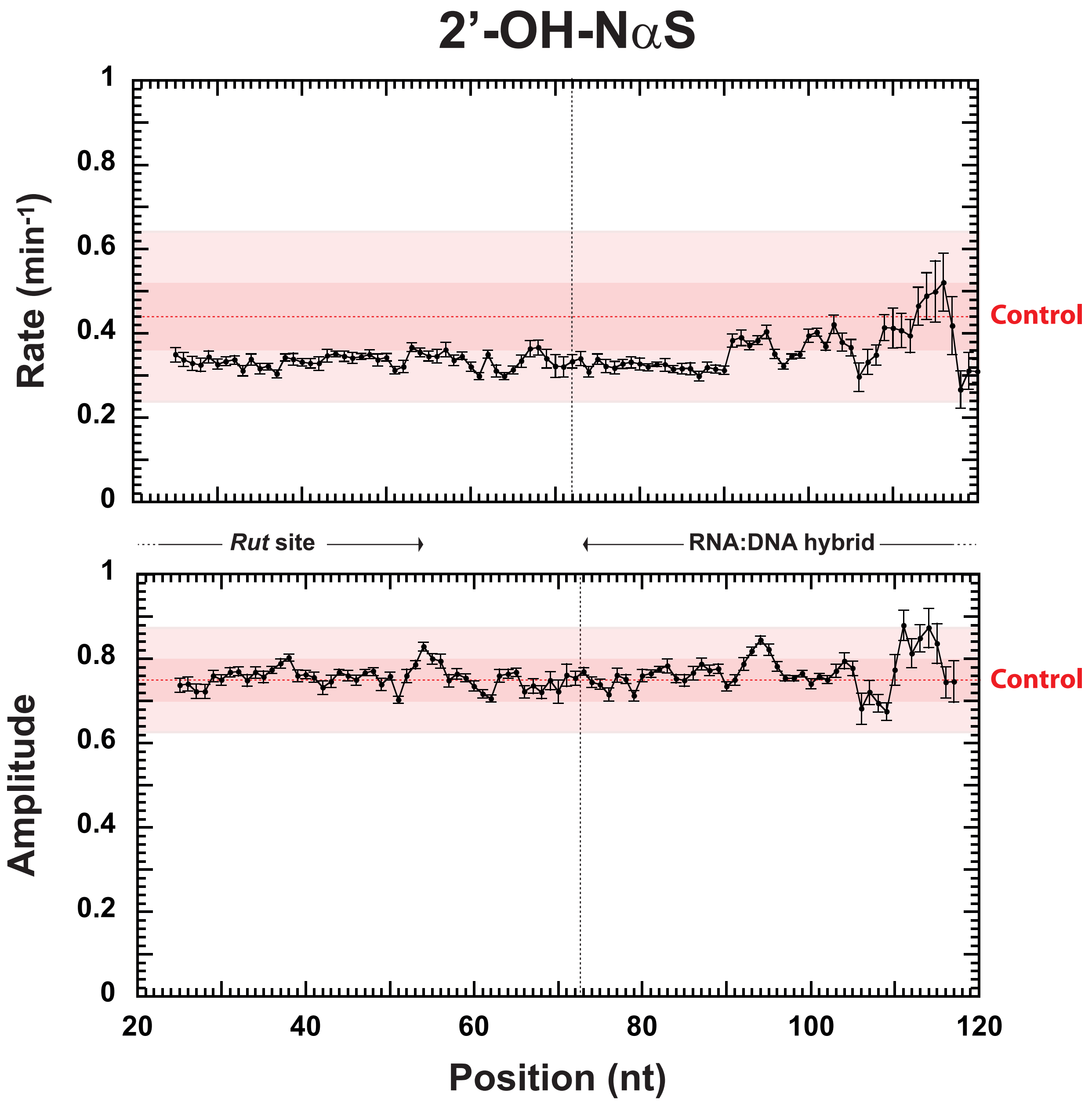

Supplementary figure 7: Control trNAIP experiments performed with parental NαS analogs.
Summary of unwinding reaction amplitudes and rates deduced from the trNAIP analysis of biotinylated RNA:DNA substrates (substrate C_{Bt}) containing parental NαS nucleotide analogs (4 independent experiments). Data points deviating by less than 1 or 2.5 SD from the 'Control' mean values fall within the dark and light red boxes (68.3% and 98.8 confidence intervals), respectively.

5′----CGAUGGUAUCAGAUCUGGAUCCUCGAGAAGCUGCGGGUACCGAGCUCGAAUUCAUCG (Sub. C)
5′----GGCACAUCGACUGAUCCACGAGCUACAGUCGAUGUCCAUGAACUACGUUGGAUGGCC (Sub. D)
5′----CCUGACAGCUGACGAAUCGAGAGAUUCCCAUGAAGUAGCUUCGAUGUACUCGGCAGG (Sub. E)
5′----GCGCUUGUAGUCUCGGAUUCUUAGCGUUGUACCUCAUCGAGUUCGAACGCACAUCGC (Sub. F)
5′----CGAGGUCAUCAGACCUGGACCCACGGGACGCUGCAGUCGACUAGGUCGACUGCUAGC (Sub. G)
80 90 100 110 120

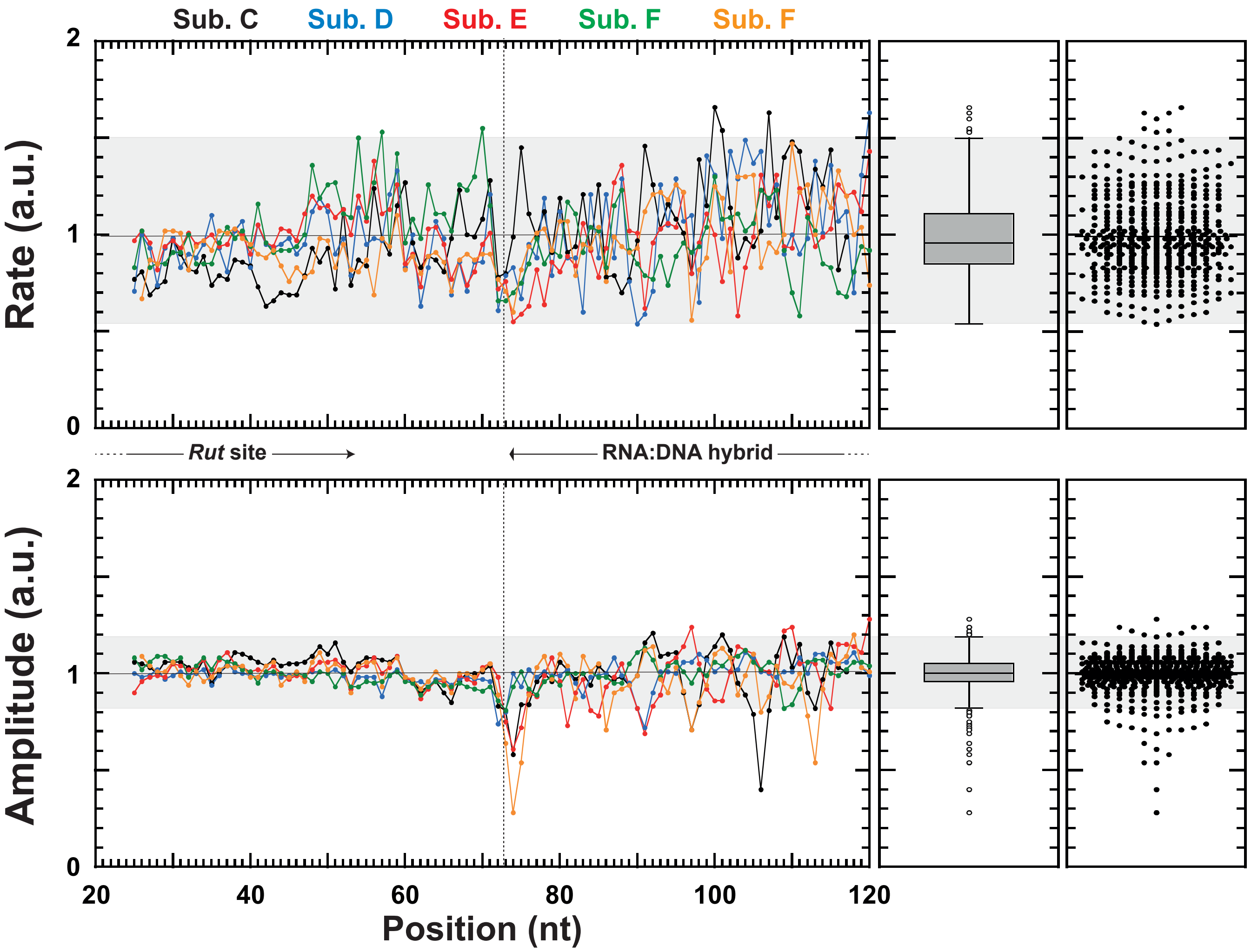

Supplementary figure S8: Summary of the trNAIP data obtained with 2'-deoxyNαS analogs and various RNA:DNA hybrid substrates.

All substrates (including substrate C) contain the same single-stranded RNA region upstream from the RNA:DNA hybrid region (only hybridized RNA regions are shown above graphs). Nucleotides in gray were not analyzed. To facilitate comparisons, amplitude and rate values were normalized relative to their series means. Dark and light grey boxes represent, respectively, the interquartile (IQR; 25th to 75h percentage) and acceptable (± 1.5 X IQR) ranges determined for the full data set (all substrates' data points shown in the rightmost dot plot) with Kaleidagraph software. Each substrate was analyzed in at least three independent trNAIP experiments. Similar to a previous NAIM study (19), no significant correlation between signal variations and nucleotide identities (or related parameter such as base pairing strength) could be uncovered.

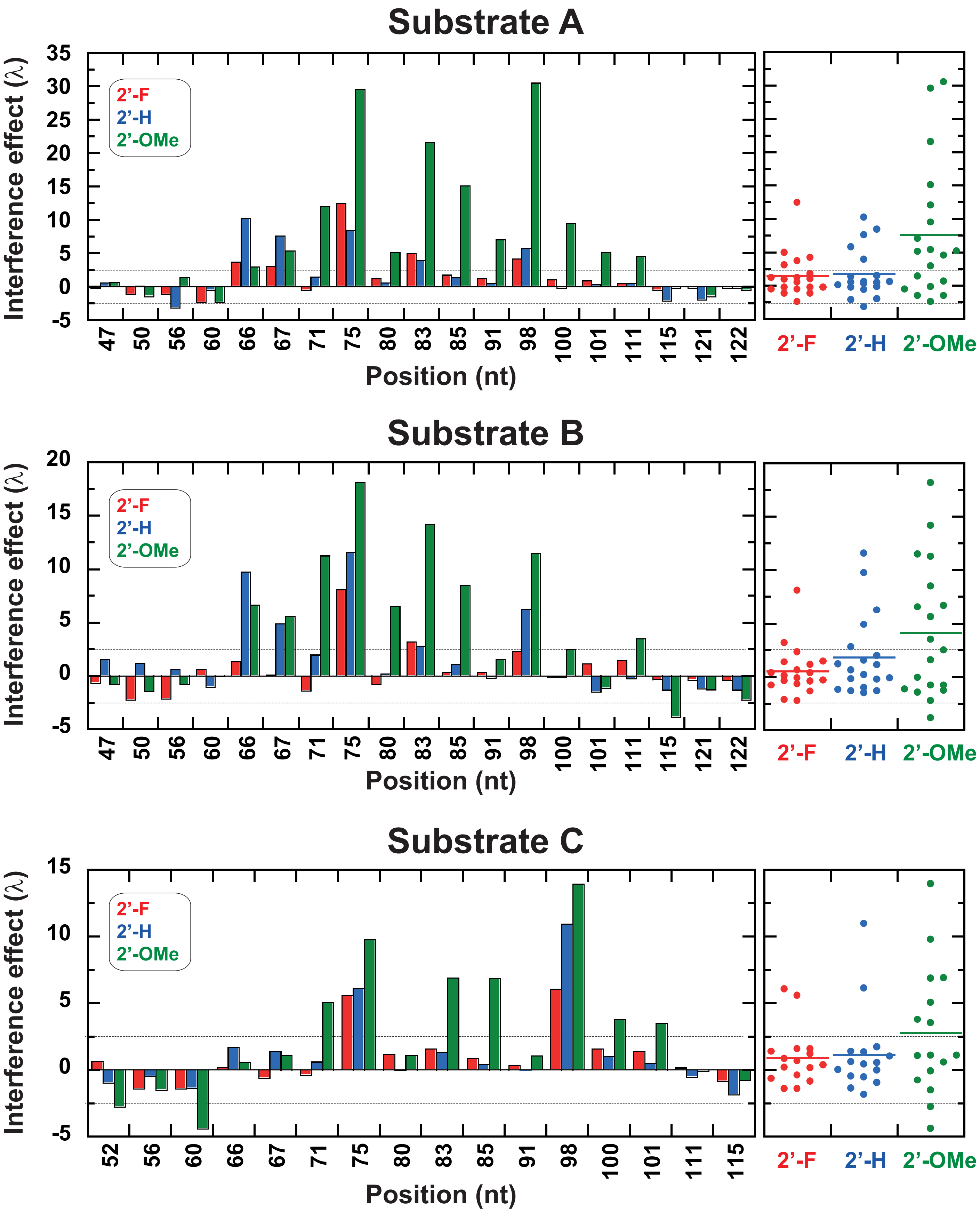

Supplementary figure S9: NAIM analysis of substrate's 2'-moieties.
Summary of 2'-Fluoro-AαS, 2'-deoxy-AαS, and 2'-O-Methyl-AαS interference effects observed upon NAIM probing of the RNA:DNA substrates shown in Figure 2A. Interference effects (λ) were normalized as described previously (19,22) with 98.8% confidence thresholds (λ = ± 2.5). To facilitate comparisons, nucleotide numbering of substrate C is used in all histograms (e.g., position 60 refers to A60 in substrate C and to A22 in substrates A and B). Horizontal bars in dot plots (right) indicate means of datasets.

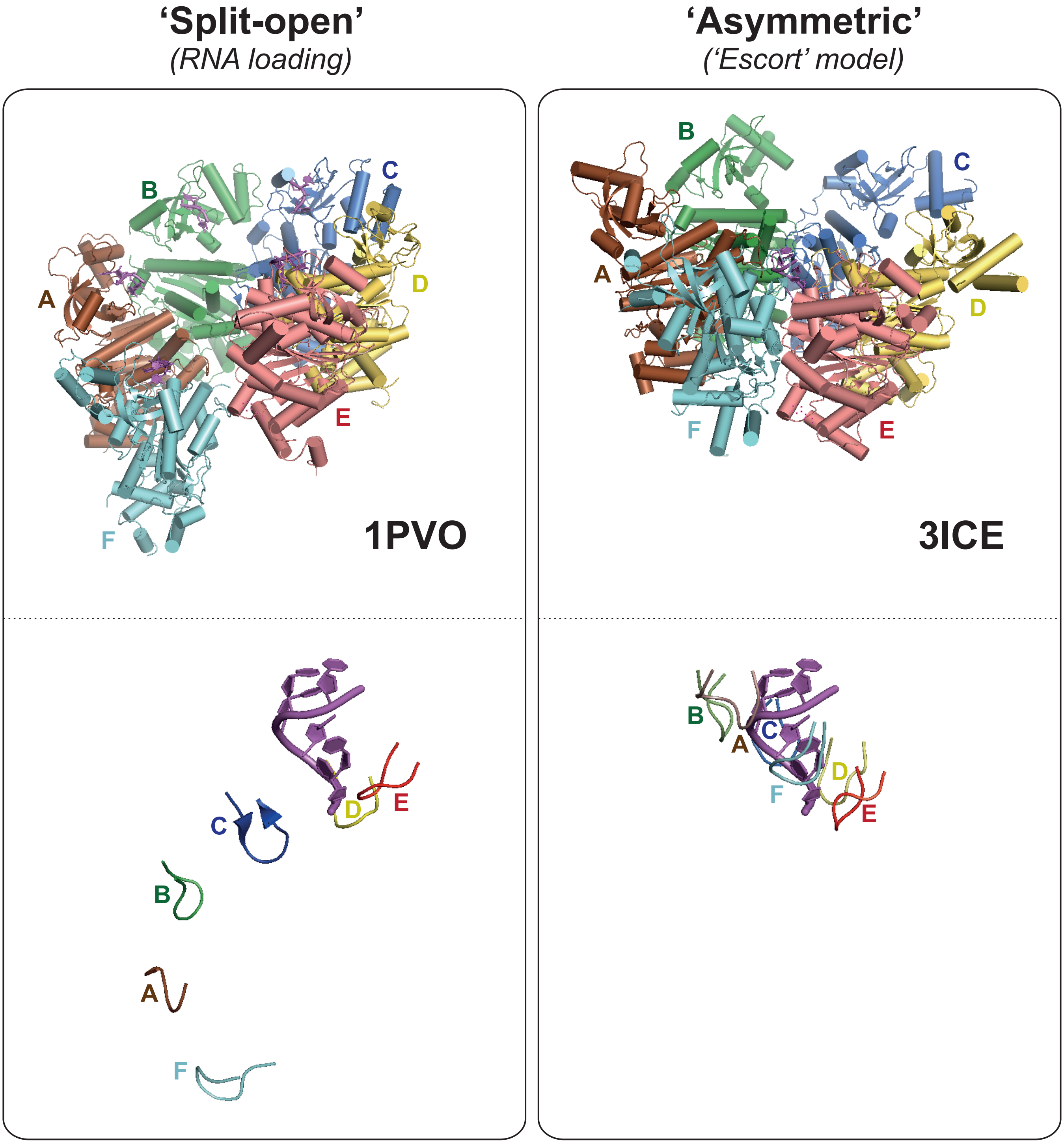

Supplementary figure S10: Extensive conformational rearrangements upon closure of the Rho ring. Comparison of the putative ‘RNA loading’ (PDB: 1PVO) and ‘translocation-competent’ (PDB: 3ICE) configurations of the Rho hexamer. Both structures were aligned through chains E before comparison. The positions of Q-loops (SBS residues 284-290) within both structures are shown in the bottom pannels with the $r(U)_6$ fragment present in the asymmetric structure (in magenta) shown in both cases to faciltate comparison. Labeling of subunits A -F is from the asymmetric structure.

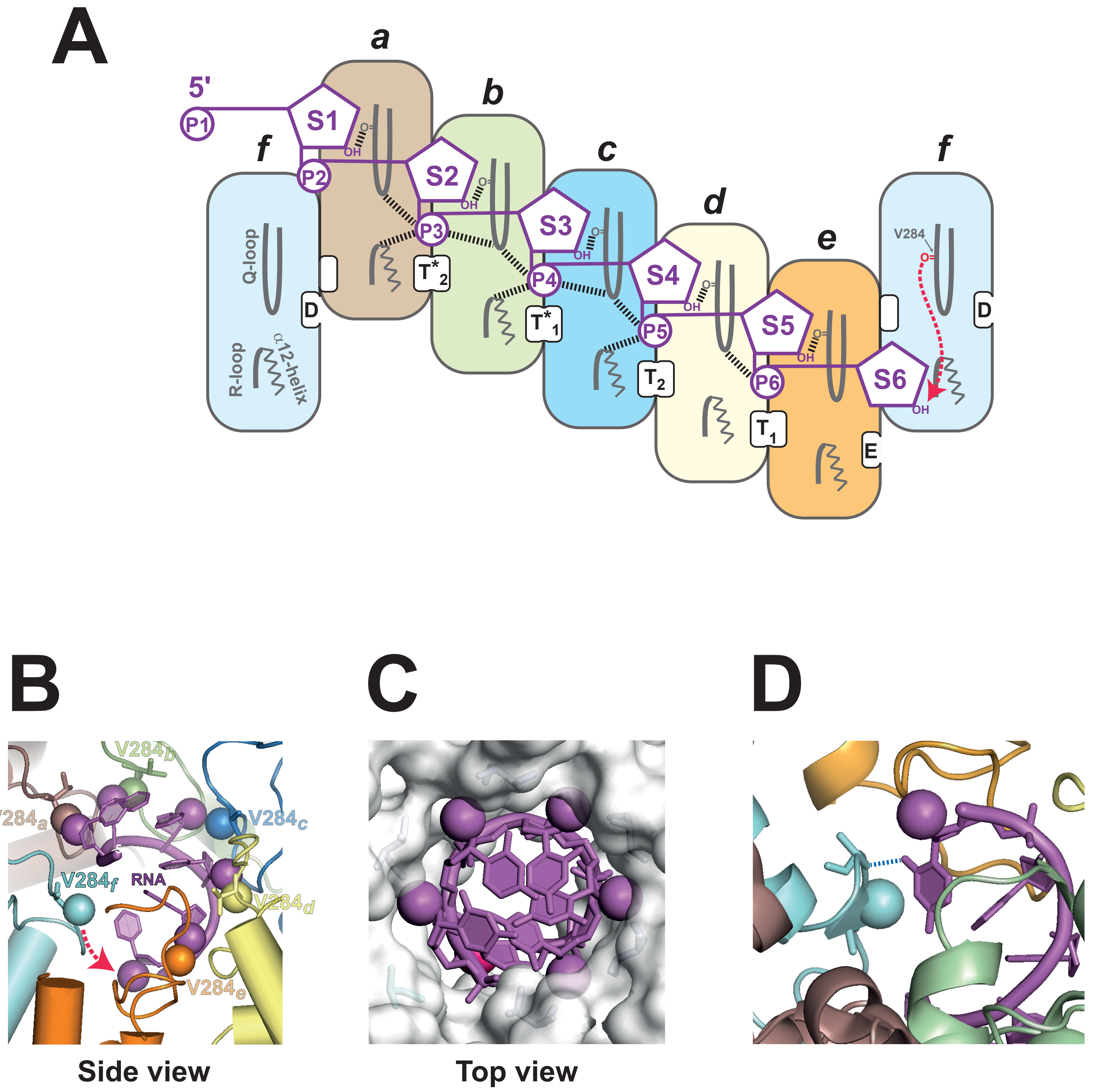

Supplementary figure S11: The SBS:RNA interaction network in the asymmetric ring structure (PDB: 3ICE). **(A)** Schematic of the SBS:RNA interaction network (adapted from ref. (14)). The ring subunits are spread flat on the page with subunit *f* shown twice. RNA sugar and phosphate moieties are numbered from the 5'-end. Note that subunit contacts to RNA base moieties are not shown here as they are likely to vary with the identities of the bases. **(B, C, and E)**: Close-up views of the RNA chain in the central channel of the asymmetric Rho ring. The 2'-OH groups on RNA (magenta) and backbone carbonyl groups of V284 residues (color code as in pannel a) on Rho are shown in sphere representation (only the 'recruiting' Val284 carbonyl and incoming 2'-OH groups are shown as spheres in pannel D). The blue dotted line in pannel D represents the interaction formed between the backbone N atom of the T286 residue on subunit *f* and the O2 atom of the 3'-uracyle residue.
